# Influence of Spatial Resolution and Compressed SENSE Acceleration Factor on Flow Quantification with 4D Flow MRI at 3 Tesla

**DOI:** 10.3390/tomography8010038

**Published:** 2022-02-10

**Authors:** Mariya S. Pravdivtseva, Franziska Gaidzik, Philipp Berg, Patricia Ulloa, Naomi Larsen, Olav Jansen, Jan-Bernd Hövener, Mona Salehi Ravesh

**Affiliations:** 1 Department of Radiology and Neuroradiology, University Medical Center Schleswig-Holstein (UKSH), Section Biomedical Imaging, Molecular Imaging North Competence Center (MOIN CC), Kiel University, 24105 Kiel, Germany; Patricia.UlloaAlmendras@uksh.de (P.U.); Jan.Hoevener@rad.uni-kiel.de (J.-B.H.); Mona.SalehiRavesh@uksh.de (M.S.R.); 2 Department of Fluid Dynamics and Technical Flows, Research Campus STIMULATE, Magdeburg University, 39106 Magdeburg, Germany; franziska.gaidzik@ovgu.de (F.G.); philipp.berg@ovgu.de (P.B.); 3 Department of Radiology and Neuroradiology, University Medical Center Schleswig-Holstein (UKSH), Kiel University, 24105 Kiel, Germany; Naomi.Larsen@uksh.de (N.L.); Olav.Jansen@uksh.de (O.J.)

**Keywords:** intracranial aneurysm, 4D flow MRI, compressed sensing, intracranial vessels, flow, partial volume effect

## Abstract

Four-dimensional (4D) flow MRI allows quantifying flow in blood vessels–non invasively and in vivo. The clinical use of 4D flow MRI in small vessels, however, is hampered by long examination times and limited spatial resolution. Compressed SENSE (CS-SENSE) is a technique that can accelerate 4D flow dramatically. Here, we investigated the effect of spatial resolution and CS acceleration on flow measurements by using 4D flow MRI in small vessels in vitro at 3 T. We compared the flow in silicon tubes (inner diameters of 2, 3, 4, and 5 mm) measured with 4D flow MRI, accelerated with four CS factors (CS = 2.5, 4.5, 6.5, and 13) and three voxel sizes (0.5, 1, and 1.5 mm^3^) to 2D flow MRI and a flow sensor. Additionally, the velocity field in an aneurysm model acquired with 4D flow MRI was compared to the one simulated with computational fluid dynamics (CFD). A strong correlation was observed between flow sensor, 2D flow MRI, and 4D flow MRI (rho > 0.94). The use of fewer than seven voxels per vessel diameter (nROI) resulted in an overestimation of flow in more than 5% of flow measured with 2D flow MRI. A negative correlation (rho = −0.81) between flow error and nROI were found for CS = 2.5 and 4.5. No statistically significant impact of CS factor on differences in flow rates was observed. However, a trend of increased flow error with increased CS factor was observed. In an aneurysm model, the peak velocity and stagnation zone were detected by CFD and all 4D flow MRI variants. The velocity difference error in the aneurysm sac did not exceed 11% for CS = 4.5 in comparison to CS = 2.5 for all spatial resolutions. Therefore, CS factors from 2.5–4.5 can appear suitable to improve spatial or temporal resolution for accurate quantification of flow rate and velocity. We encourage reporting the number of voxels per vessel diameter to standardize 4D flow MRI protocols.

## 1. Introduction

Blood flow is altered in a variety of intracranial diseases, such as arterial stenosis [1,2], intracranial aneurysms [3,4], and vascular malformations [5]. The study of hemodynamics in general can potentially improve diagnostic capabilities, therapeutic planning, and patient follow-up.

Four-dimensional (4D) phase-contrast magnetic resonance imaging (4D flow MRI) is a noninvasive technique and can be used to quantitatively and qualitatively analyze the hemodynamics of blood vessels [6]. It can provide time-resolved 3D visualization of complex flow patterns and quantify flow rates, velocities, and more advanced parameters, such as wall shear stress (WSS) and oscillatory shear index (OSI) [7,8,9,10]. Four-dimensional flow MRI is widely used for cardiac examinations, where the diameter of the arteries (e.g., thoracic or abdominal aorta) is relatively large. In neurovascular applications, 4D flow MRI has been used primarily for research purposes to study intracranial aneurysms, arteriovenous malformation, and Alzheimer’s disease, among others [11]. Furthermore, neurovascular 4D flow MRI can potentially be used to monitor the effect of aneurysm therapy [12,13,14].

Although the number of neuroradiological applications for 4D flow MRI is increasing [15], the technique is rarely used as part of standard clinical care, as compared to the cardiovascular applications. Factors impeding widespread clinical use of 4D flow MRI include insufficient spatial resolution to assess the flow in small vessels and velocities near the vessel wall, long examination times, and high sensitivity to velocity-encoding parameters (Venc) [16]. Similar to reconstruction, quantification, and visualization of velocity, these datasets have needed to be evaluated offline, too, often by physicists. Nowadays, the vendors offer online reconstruction of the datasets, as well as basic data post-processing, for example, flow peak velocity, but not kinetic energy or vorticity [17].

Nevertheless, some initial studies have demonstrated that certain types of 4D flow MRI examinations can be obtained in about 2–8 min [18,19,20,21]. Therefore, we believe that implementation in clinical routine is inevitable. First, however, 4D flow MRI protocols need to be standardized and awareness of confounders is essential to achieve a broad clinical application.

Previously, velocity and flow measurements from 4D flow MRI were compared with 2D flow MRI [22,23], Doppler ultrasound [24], particle imaging velocimetry (PIV) [25], and numerical simulations based on computational fluid dynamics (CFD) [26,27]. However, studies focusing on the impact of 4D flow MRI sequence parameters, such as spatial resolution, on flow quantifications are limited, especially for neurovascular applications [22,28].

Thus, we aimed to study the effect of spatial resolution in combination with acceleration on flow rates and velocity measured using 4D flow MRI. Three voxel sizes (0.5, 1.0, and 1.5 mm^3^) and four compressed SENSE (CS) acceleration factors (2.5, 4.5, 6.5, and 13) were considered. We investigated the matter in vitro using silicone tubes of different diameters (2, 3, 4, and 5 mm) and a patient-derived 3D-printed aneurysm model. The results were compared to flow sensor, 2D flow MRI, and CFD simulations.

## 2. Materials and Methods

### 2.1. Flow Models and Circulation Setup

**Figure 1:**
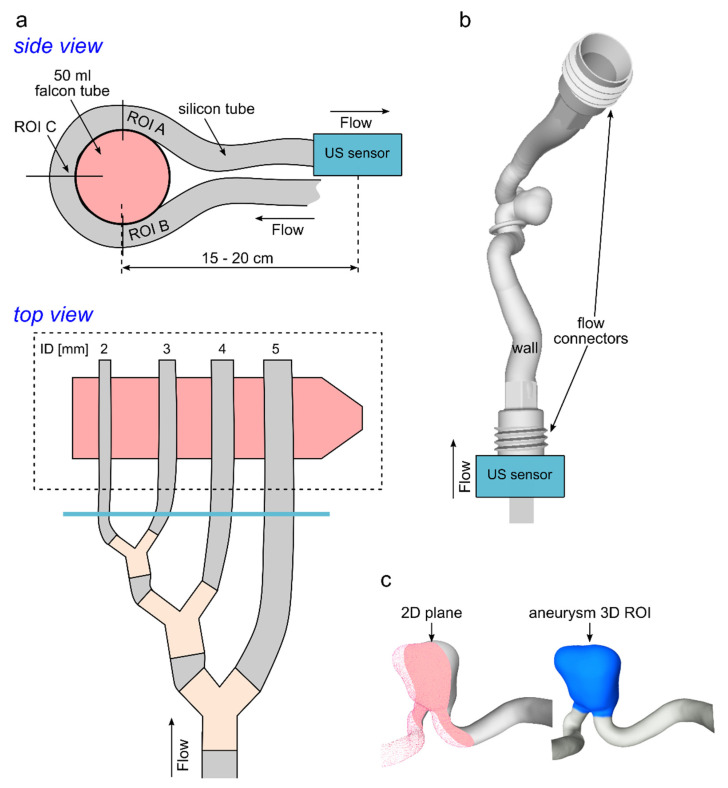
Schematic view of model 1 ((**a**) silicone tubes) and model 2 ((**b**) patient-derived aneurysm). (**a**) Silicone tubes were wound around a falcon tube (**a** top) and connected sequentially to the pump (**a** bottom). A US sensor was placed at the outflow of the tubes (blue line). The flow was analyzed at the plane’s ROI A-C. (**b**) Volume rendering of the patient-derived vascular model. A US sensor was placed at the inlet of the model. (**c**) The velocity field was visualized at the 2D evaluation plane (**c** left) and calculated in the aneurysm 3D ROI (**c** right).

A set of silicone tubes with inner diameters (ID) in the range of 2–5 mm was used to create a model of tortuous neurovascular vessels (Figure 1a). To do so, tubes were wound around a 50-mL falcon tube. Chosen diameters cover the dimensions reported for human intracranial arteries, such as internal carotid artery (ICA, ID = 3.6 ± 0.9 mm), middle carotid artery (MCA, ID = 2.5 ± 0.6 mm), anterior cerebral artery (ACA, ID = 1.8 ± 0.6 mm), basilar artery (BA, ID = 3.3 ± 1.0 mm), and posterior cerebral arteries (PCA, ID = 2 ± 0.5 mm) [29]. Tubes were connected with a pulsatile pump (PD-1100, BDC Laboratories, Wheat Ridge, CO, USA). The glycerol-water mixture (40/60% by volume) circulated in the flow setup. The testing fluid had a viscosity of 3.72 cP according to Segur et al. [25], which corresponds to the viscosity of blood (3.5 cP [26]). Pulsatile flow at mean flow rates of 0.6, 1.2, 2.3, and 3.1 mL/s was applied to tubes with an ID of 2–5 mm, respectively, which correspond to the flow observed in vivo in the ICA (4.3 ± 0.8 mL/s), MCA (2.4 ± 0.5 mL/s), BA (2.4 ± 0.7 mL/s), and PCA (0.9 ± 0.2 mL/s) [30]. The flow peak velocity was about 60 cm/s in all four tubes. Therefore, the same flow velocity-encoding parameter (60 cm/s) was set during all MRI measurements (details below). The flow in the tubes was measured before MRI experiments one by one with a transonic flow sensor (US sensor, Transonic System Inc., Ithaca, NY, USA) located 10 cm downstream from the model outlets. The flow setup proved to be stable over at least 3 h (details in Figure S1).

A patient-specific aneurysm model was designed and 3D printed (Form 3, Formlabs Inc., Somerville, MA, USA) in-house [31] (Figure 1b). Aneurysm geometry was segmented from 3D rotational angiographic data of a patient with a 17.5-mm infraophthalmic extradural internal carotid artery (ICA) aneurysm. A mixture of methacrylic acid esters and a photoinitiator (Clear Photoreactive Resin, Formlabs Inc., Somerville, MA, USA) was used for 3D printing of an aneurysm model with a rigid wall. The inlet and outlet flow and pressure were measured approx. 10–15 cm apart from the model inlet and the outlets with the US flow and pressure (PRESS-N-000, PendoTech, Princeton, NJ, USA) sensors, respectively. For simplicity, tap water was circulated at the flow setup at a flow rate of 3.8 mL/s (Ismaltec MCP Standart, Cole Parmer, Vernon Hills, IL, USA). The chosen flow rate corresponds to a flow observed in vivo [30]. The velocity field was analyzed at the 2D plane (Figure 1c, left) and over the 3D volumetric aneurysm ROI (ROI, Figure 1c, right).

In addition, 3% (weight/weight) agarose gel was poured around the silicone tubes and aneurysm model before the MRI experiment to increase the overall MR signal of the in vitro models and to eliminate the air–tissue interface artifacts. Reinforced, polyvinyl chloride, braided tubing with an inner diameter of 6 mm and 2 m in length was used to connect the models to the pump placed outside of the MRI room.

All measurements were acquired at room temperature (21 °C). The silicone tubes and aneurysm model were supplied with different pumps due to the unavailability of the same circulation setup during each experiment. However, this fact should not impact the general applicability of these results.

### 2.2. Magnetic Resonance Imaging

A whole-body 3 T MRI clinical system equipped with a 32-channel volume head coil (Ingenia CX, R5 V6.1, Philips Healthcare, Best, the Netherlands) was used for all MRI experiments. The imaging protocol comprised: time-of-flight (TOF), 2D flow, and 4D flow MRI. All sequences were accelerated by using a compressed SENSE technique based on sensitive encoding (CS-SENSE) implemented by the vendor (Philips) [32,33]. CS-SENSE uses incoherent undersampling of variable density together with iterative (non-linear) reconstruction. CS-SENSE consists of a mix between compress sensing theory [34] and SENSE technology (parallel imaging technique) [35].

Both 2D and 4D flow MRI were acquired with the spoiled gradient-echo sequence with Cartesian sampling [15]. The maximum velocity was estimated by performing a series of 2D flow MRI measurements with varied velocity-encoding parameters (Venc) in the range from 30–150 cm/s. The final Venc values were chosen to be 10% above the maximum detected velocity and were equal to 60 and 80 cm/s for the experiment with silicone tubes and an aneurysm model, respectively, for both 2D and 4D flow MRI. One planar velocity encoding was used for 2D flow MRI and three velocity encoding directions were used for 4D flow MRI. The cardiac cycle was equal to 800 and 816 ms for the silicone tubes and aneurysm model, respectively, and 24 measurement points over a cardiac cycle were obtained. The resulting temporal resolution was 33–34 ms (acquired heart phases were set at 100%). Four-dimensional flow MR images were acquired using a balanced symmetric four-point phase-contrast velocity-encoding scheme (Hadamard) [36]. Acquisition planes of the 2D flow MRI were positioned perpendicular to the flow direction, as shown in Figure 1a (ROI A–C).

Isotropic voxel size (0.5, 1.0, and 1.5 mm^3^) and CS acceleration factor were varied by the operator; all other parameters were kept the same or were set automatically by the system. TR and TE were automatically set to the shortest values allowed by the MRI system. MRI protocol parameters are summarized in Table 1.

Silicone tubes were imaged by 2D flow (P1, Table 1) and 4D flow MRI (P2–P4, Table 1). Acceleration factors of 4D flow MRI were 2.5, 4.5, 6.5, and 13, and the 4D flow MRI examination time varied from 57.5 to 1.5 min with increasing acceleration factor.

The aneurysm flow model was imaged by 2D flow (P1, Table 1), 4D flow (P5–P7, Table 1), and TOF MRI (P8). Acceleration factors of 4D flow MRI were 2.5, 4.5, and 6.5, and the 4D flow MRI examination time was reduced from 73.2 to 3.2 min with an increasing acceleration factor.

### 2.3. Time-Resolved Hemodynamic Simulations

To enable a comparison between the measured aneurysm velocity field and the highly resolved in silico velocity results, image-based blood flow simulations were carried out. The numerical geometry was segmented from the TOF MRI data in vitro. Before performing the corresponding simulation, the model was spatially discretized using STAR-CCM+ 2020.01 (Siemens Product Lifecycle Management Software Inc., Plano, TX, USA) with a cell base size of Δx = 0.1 mm, resulting in a total number of 5.5 million cells (polyhedral and prism layers). The flow rate measured using a flow sensor was applied to the inlet cross-section, and the outlet boundary condition was set to the pressure values provided by the pressure sensor placed in the aneurysm outlet. Similar to the in vitro model, all vessels were assumed to be rigid, and no-slip boundary conditions were applied. Finally, identical fluid properties (water) were defined for density and viscosity. The maximum Reynolds number (Re) was calculated with the mean velocity (u = 20 cm/s) and diameter (ID = 0.5 cm) of the ICA as Re = (u × ID × ρ)/μ, [37], with the water viscosity and density of μ = 0.890 cP and ρ = 997 kg/m^3^, respectively. This resulted in Re ≈ 1120; therefore, laminar flow conditions were considered.

To obtain a periodic solution, the time-dependent blood flow simulation constitutes three cardiac cycles (time step size Δt = 0.001 s). However, the first two cycles were discarded, and only the last one was included in the analysis. The final post-processing of the simulation data was carried out in EnSight 10.2 (ANSYS Inc., Canonsburg, PA, USA).

### 2.4. Data Processing

The 2D and 4D flow MR images were reconstructed on the MRI console. Subsequently, the 2D and 4D flow MRI data were pre-processed as follows (GTflow, Version 3.1.12, Gyrotools, Switzerland):

1. The linear offset phase correction was conducted on each slice individually to correct for the presence of eddy currents. The fit was calculated at the reference heart phase (at the peak flow time) and then applied to all heart phases. A phase correction to compensate for concomitant gradients (Maxwell terms) and geometry correction to compensate for inhomogeneities of the main magnetic field and non-linearity of the gradient fields was performed on MR systems as part of the standard phase-contrast MR image reconstruction.
2. Velocities in voxels outside of the flow lumen were nulled based on a magnitude intensity threshold.
3. The data were inspected against phase-aliasing and manually corrected if necessary.

Next, the following processing steps were performed:

1. ROIs were created manually on MRI magnitude data. First, contours around the tube’s lumen were drawn using a b-spline curve (feature in GTflow) on 2D flow MRI. Note that the 2D flow MRI acquisition planes are already perpendicular to the flow direction. Next, the resulting 2D flow ROIs were translated to the 4D flow MRI data, ensuring identical placement of ROIs on the 2D and 4D flow datasets.
2. In a given ROI, the flow of all voxels was summed up for each time point $f\left(t\right)=\sum_{i}f_{i}\left(t\right)$, where *i* indicates the voxel and *t* the temporal point. The flow was spatially averaged over ROI A-C, as follows: $f\left(t\right)=\frac{1}{3}\left(f{\left(t\right)}_{ROI\;A}+f{\left(t\right)}_{ROI\;B}+f{\left(t\right)}_{ROI\;C}\right)$.
3. The number of voxels per ROI diameter (nROI) was calculated to obtain a measurement not depending on the voxel size and vessel diameter as $nROI=\frac{ROI\;diameter\;\left[mm\right]}{1D\;voxel\;size\;\left[mm\right]}$.
4. The time-dependent difference between flow values obtained with 4D and 2D flow MRI was calculated as $difference\;\left(t\right)\;\left[\%\right]=\frac{f{\left(t\right)}^{4D\;flow}-f{\left(t\right)}^{2D\;flow}\;}{mean(flow\left(2D\;flow\right)}\cdot 100\%$. Similarly, the difference between flow values obtained with 4D flow MRI and US sensor was calculated.
5. The normalized root-mean-square (RMS) error was used to assess the accuracy of flow quantification. RMS was calculated as the sum of squared differences between 2D and 4D flow MRI data over the time steps and normalized by the time-averaged flow acquired with 2D flow MRI: $RMS=\frac{\sqrt{\sum_{t}^{Num}{\left(f{\left(t\right)}^{4D\;flow}-f{\left(t\right)}^{2D\;\mathrm{flow}\;}\right)}^{2}}/Num}{mean\;flow\;\left(2D\;flow\right)}$, where *t* indicates the temporal measurement point and *Num* is the number of temporal points. Similarly, RMS between flow values obtained with 4D flow MRI and US sensor was calculated.
6. The velocity magnitude for each voxel in the aneurysm 3D ROI (Figure 1d, right) was calculated and averaged over time $v_{i}^{mag}=\frac{\sum_{t}^{Num}\sqrt{{\left(v_{i}^{x}\;\left(t\right)\right)}^{2}+{\left(v_{i}^{y}\left(t\right)\right)}^{2}+{\left(v_{i}^{z}\left(t\right)\right)}^{2}}}{Num}$, where *i* indicates voxel, *t* temporal point, and *Num* the number of temporal points. Aneurysm velocity distributions were presented using histograms similar to Schnell et al. [38].
7. Time-averaged velocity magnitude in the evaluation plane across the aneurysm was visualized pixel-wise on a color-coded representation (MATLAB R2019a, MathWork, Natick, MA, USA).
8. Repeatability of flow measurements with 2D flow MRI was assessed with repeatability coefficient (RC) as follows: (1) 2D flow MRI was measured five times in parental vessel and at the aneurysm sac; (2) RC was calculated for each time point using the equation adapted from Raunig et al. [39] $RC\left(t\right)=1.96*\sqrt{2SD{\left(t\right)}^{2}}/f^{mean}\left(t\right)*100\%$, where *SD* is the standard deviation and $f^{mean}$ is the mean flow rate over five measurements; (3) time-depended RC were time-averaged as $RC=\sum_{t}^{Num}RC\left(t\right)/Num$, where *t* indicates the temporal measurement point and *Num* is the number of temporal points.

Here, we mostly aimed to assess flow in silicone tubes measured with 4D flow MRI against 2D flow MRI. A detailed comparison to the US sensor is presented in the paper supplement. In the main manuscript, we focus on the comparison with 2D flow MRI, because the location of 4D and 2D flow MRI were identical while the US sensor was located at a considerable distance between MR imaging volume. The distance between flow measurement positions has no impact on total flow at the tubes in the closed circuit. However, it might impact individual time-depended flow values, for example making the flow curve flatter, but keeping the total flow volume the same. As we want to assess time-depended flow curves, we limited the comparison of 4D flow MRI at the main text to the method that can be performed at the same location, namely 2D flow MRI here.

### 2.5. Statistical Analysis

Normality (Gaussian distribution) was tested for all variables (flow rate, flow difference) using the Shapiro–Wilk test (Tables S1 and S2) [40]. The median and range of variables were reported as summary statistics for all variables due to the severe skewness of the respective distributions. Spearman’s rank correlation coefficient (rho) and linear fit coefficient were calculated to assess the similarity of flow rate values obtained by US sensor and 2D flow, US sensor and 4D flow MRI, 2D and 4D flow MRI measured in silicone tubes, and to assess the relationship between flow differences and RMS on nROI. A two-sided paired Wilcoxon signed-rank test was conducted for the flow rate values measured with a US sensor, 2D flow MRI, and 4D flow MRI in silicone tubes to assess the statistical difference between datasets. The same test was conducted to assess the significance of flow differences from 5 and 10% of median flow rate values measured with US sensor and 2D flow MRI. To assess the impact of the CS factor on RMS and median flow differences, the Kruskal–Wallis test was performed where CS was as a grouping factor. The impact of spatial resolution on velocity distribution in an aneurysm model was assessed with the Kruskal–Wallis test too, where voxel size was as a grouping factor. A two-sided Wilcoxon rank-sum test was conducted to compare velocity distribution obtained using CS = 2.5 with CS = 4.5 and 6.5 for each spatial resolution independently. The level of significance for all tests was set as *p*-value = 0.05. MATLAB R2020a (MathWork, Natick, MA, USA) was used for statistical analysis. The graphic representation was generated using Microsoft Excel 2016 (Microsoft Corporation, Redmond, WA, USA).

## 3. Results

### 3.1. Flow in Silicone Tubes

**Figure 2:**
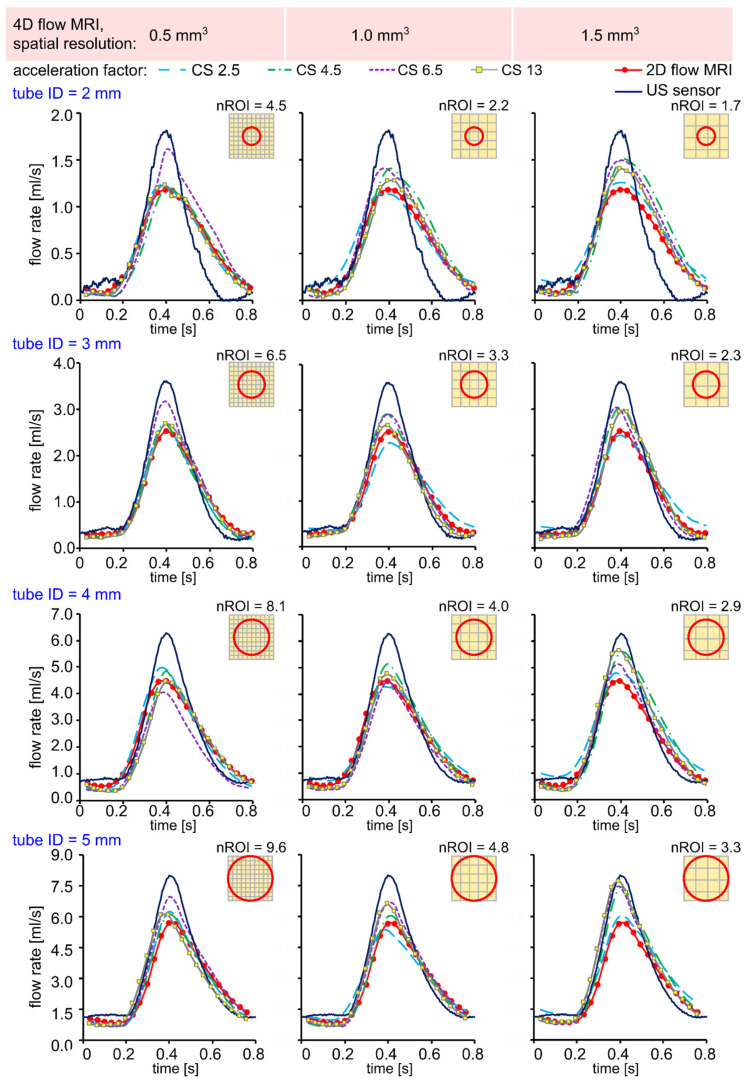
Time-resolved flow rate curves measured with US sensor, 2D flow MRI, and 4D flow MRI in silicone tubes with an inner diameter (ID) of 2, 3, 4, and 5 mm, respectively. The flow rates were calculated in three ROI A-C, as shown in Figure 1, and then averaged. Qualitatively, the peak flow values were overestimated for lower resolution 4D flow MRI data in comparison to 2D flow MRI.

The flow patterns obtained using the US sensor and 2D flow MRI were qualitatively similar to those obtained with 4D flow MRI for all voxel sizes and CS acceleration factors (Figure 2).

First, as a sanity check, we compared flow measured with US sensor and the 2D flow MRI. Data were strongly correlated (rho = 0.96, Figure S2). Median flow difference was 12.94, −2.97, −2.90, and −6.76% for tube ID = 2–5 mm, respectively. Only for tube ID = 5 mm, median flow difference was significantly different from 0% (*p* = 0.03), but not different from 5% (*p* = 0.61). Therefore, the flow measured with US sensor and 2D flow MRI was in good agreement. Furthermore, we report the results of the comparison between 4D and 2D flow, while the quantitative comparison between flow obtained with a US sensor and 4D flow MRI is presented in a supplement to this paper (Figures S2 and S3, Tables S3 and S4).

**Figure 3:**
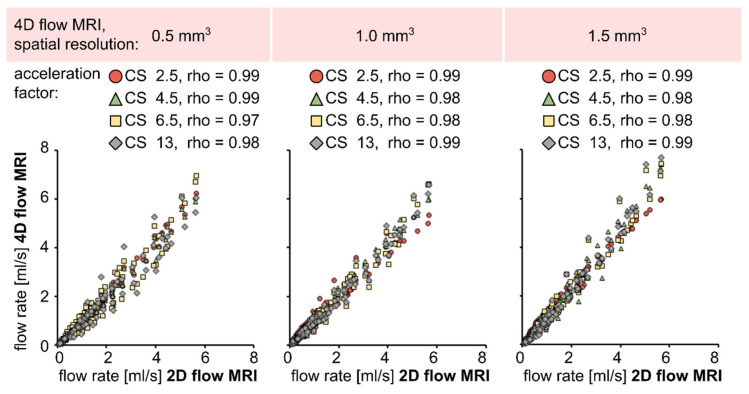
Scatter plots and Spearman-rank correlation coefficient between time-resolved net flow rate values obtained by 2D and 4D flow MRI in silicone tubes. A strong correlation was observed for all voxel sizes and acceleration factors (rho > 0.97).

Quantitatively, a strong correlation (rho > 0.97) was observed between 2D and 4D flow MRI for all voxel sizes and CS factors (Figure 3). A strong correlation (rho > 0.94) was also observed between US sensor and 4D flow MRI (Figure S3). In addition, a linear fit was performed on 2D and 4D flow MRI flow values (R^2^ > 0.96, Table 2). The linear slope was close to 1 for 0.5 and 1 mm^3^ voxel size, regardless of the CS factor. For a voxel size of 1.5 mm^3^, the linear slope was close to 1 only for CS = 2.5. At higher acceleration factors (CS = 4.5–13), the flow values with 4D flow MRI were overestimated in comparison to 2D flow MRI (1.21 < slope < 1.29).

**Figure 4:**
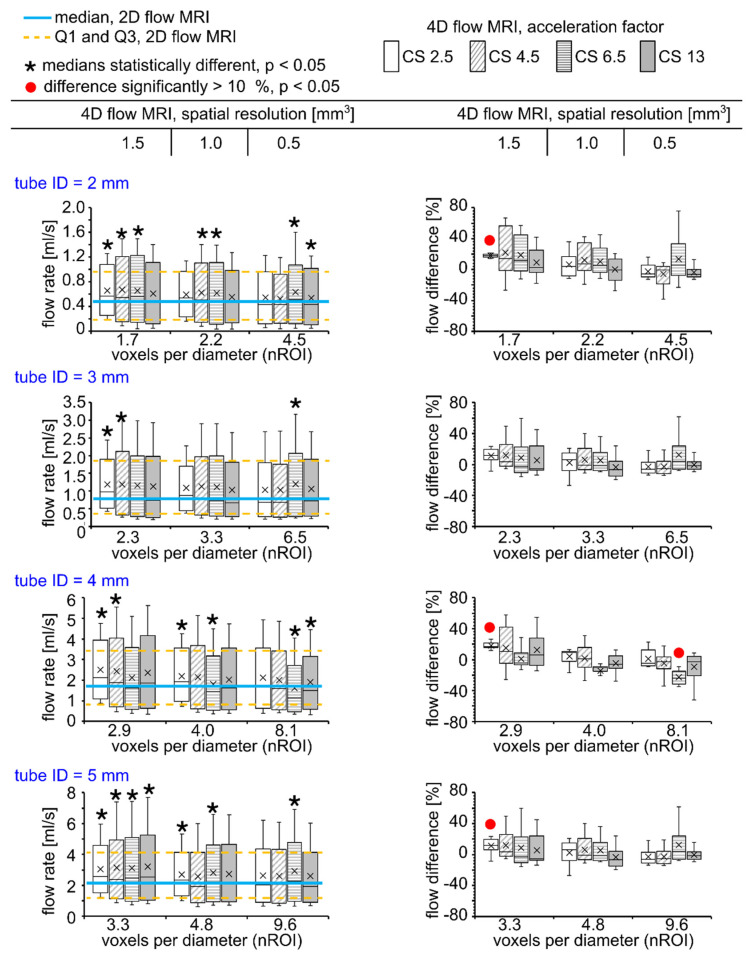
Box plots representing flow values obtained with 4D flow MRI (left) and flow differences calculated between 4D and 2D flow MRI (right). A voxel size of 1.5 mm^3^ and CS = 2.5 and 4.5 resulted in statistically different flow median values (*p* < 0.001, star sign, left, a two-sided paired Wilcoxon signed-rank test). However, the difference was significantly higher than 10% only for four datasets (*p* < 0.001, red circle, right, a two-sided paired Wilcoxon signed-rank test).

Overall, the flow was overestimated in all experiments by an average of 1.33 ± 8.31% and 0.24 ± 10.73% in comparison to 2D flow MRI and US sensor, respectively, for all voxel sizes and CS acceleration factors used for 4D flow MRI. A two-sided paired Wilcoxon signed-rank test revealed the statistical difference between flow values measured with 4D and 2D flow MRI for almost half of the used combinations of spatial resolution and CS factors (Figure 4 left). For example, the flow median was statistically overestimated by voxel size equal to 1.5 mm^3^ and CS = 2.5 and 4.5 for all tubes (*p* < 0.05). Summary statistics of flow rate values for each tube ID, voxel size, and CS factor are summarized in the supporting information (Table S3).

Flow difference between 4D and 2D flow ranged from –23.75% (tube ID = 4 mm, voxel size = 0.5 mm^3^, CS = 6.5) to +18.31% (tube ID = 2 mm, voxel size = 1.5 mm^3^, CS = 2.5). However, the median of flow difference calculated between 4D and 2D flow MRI was statistically higher than 10% only for voxel size of 1.5 mm^3^ and CS = 2.5 for tube ID = 2, 4, and 5 mm (*p* < 0.001), and for a voxel size of 0.5 mm^3^ and CS = 6.5 for tube ID = 4 mm (*p* < 0.001, Figure 4 right). Summary statistics of flow differences for each tube ID, voxel size, and CS factor is summarized in supporting information (Tables S4 and S5). Flow values and flow differences were obtained by 4D flow MRI in comparison to US sensor presented in Figure S4.

**Figure 5:**
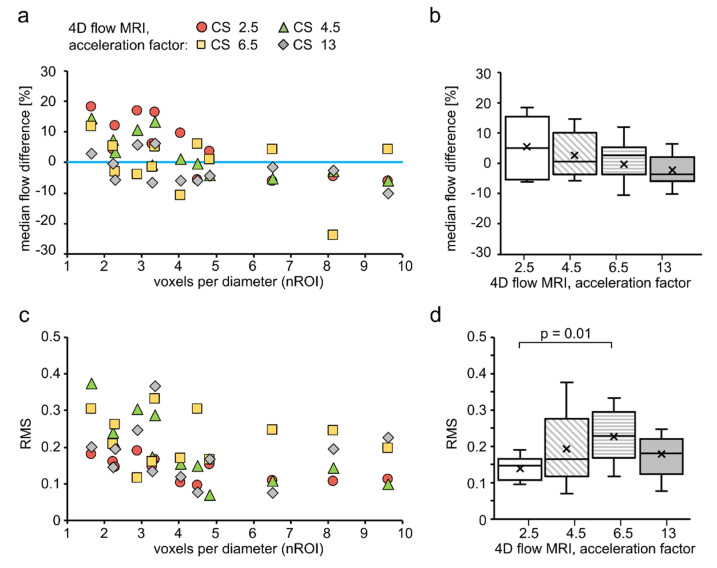
Scatter (left) and box plots (right) for experiments performed on silicone tubes. (**a**) The median difference between flow rate values measured with 4D and 2D flow tended to decrease with increasing voxel number per diameter. (**b**) Median flow difference tended to decrease with an increased CS factor. (**c**) RMS tended to decrease with increasing voxel number per diameter. (**d**) RMS tended to increase with increased CS factor from 2.5 to 6.5. RMS obtained with CS = 2.5 was significantly different from CS = 6.5. For creating box plots in (**b**,**d**), RMS and flow difference values, obtained by 4D flow MRI with various nROIs but with the same CS factor, were joined together to make one dataset. Therefore, the dependency of data on nROI was excluded.

There might be an interplay between the size of the tube diameter and voxel size. Therefore, we constructed a dimensionless surrogate representing both voxel size and tube, termed here voxel number per vessel diameter (nROI). Increased nROI leads to a decreased median flow difference (Figure 5a). A weak correlation was observed between median flow differences and nROI (rho = −0.55. *p* < 0.01) when all CS factors were considered. A strong negative correlation was found for individual acceleration factors CS = 2.5 and 4.5 between median difference and nROI, rho = −0.81 (*p* = 0.02) and rho = −0.85 (*p* < 0.01), respectively, while no correlation was present for CS = 6.5 and 13 (rho < −0.44). Therefore, nROI plays the dominant role in the flow accuracy when CS ≤ 4.5, while when CS ≥ 6.5, some other factors might play a greater role. The trend of decreased median flow differences was observed with increased CS factor (Figure 5b); however, no significant impact of CS factor was found according to the Kruskal–Wallis test (*p* = 0.18).

Next, we assessed RMS value to evaluate an absolute flow error. Overall, RMS was equal by an average of 0.18 ± 0.08 for all voxel sizes and CS acceleration factors used. RMS ranged from 0.07 (nROI = 4.8, CS = 4.5) to 0.38 (nROI = 1.66, CS = 4.5). As a result, we observed that increased nROI leads to decreased RMS values (Figure 5c). If more than 7 voxels are used, the RMS value tends to plateau. No correlation between RMS and nROI was found when the RMS values from all CS factors were considered (rho = −0.38, *p* = 0.007). However, for individual CS factors = 2.5 and 4.5, a strong negative correlation was found between RMS and nROI, rho = −0.63 (*p* = 0.03) and rho = −0.87 (*p* < 0.01), respectively. While for CS factors = 6.5 and 13, no correlation was found (rho < −0.15). Here, again nROI plays the dominant role in the flow accuracy when CS ≤ 4.5, while when CS ≥ 6.5, some other factors might play a greater role. The trend of increased RMS was observed with increased CS factor from 2.5 to 6.5 (Figure 5d). Statistical difference between CS = 2.5 and CS = 6.5 was found according to Kruskal–Wallis test (*p* = 0.01).

RMS and median flow difference depending on nROI calculated for 4D flow MRI in comparison to the US sensor is presented in Figure S5.

**Figure 6:**
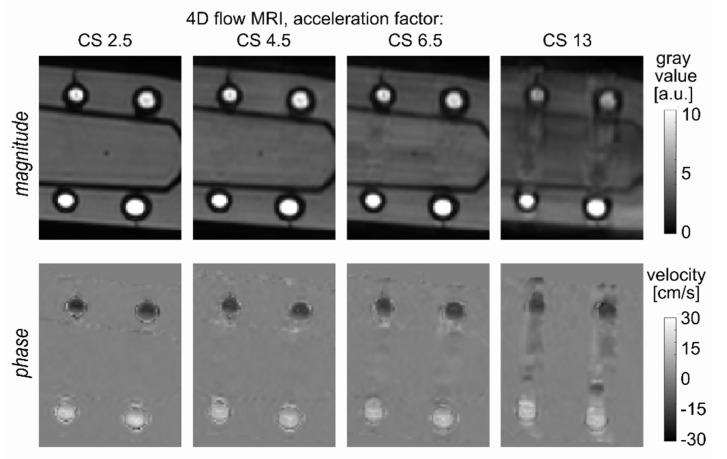
A representative cross-section of the silicone tubes with a diameter of 4 and 5 mm that were imaged with 4D flow MRI. The sequence was accelerated factors of 2.5-, 4.5-, 6.5-, and 13 (from left to right), and an isotropic voxel size of 1 mm^3^ was used. Note the severe image distortion when an acceleration factor of 13 is applied. Phase image with velocity encoding in foot to head direction, which is collinear with a flow direction, is shown.

The effect of high CS factors = 6.5 and 13 is not clear based on our results, although the trend of changing flow differences and RMS values was observed with an increasing CS factor. The flow curves still were very similar to each other. However, severe distortions were apparent in the MRI magnitude, and phase images obtained with 4D flow MRI with CS = 13 (Figure 6). The robustness of flow curves to the acceleration factor is likely due to a great difference in the voxel intensity originating from the silicone tube wall and lumen. Thus, the definition of the flow region of interest was not affected by signal distortion.

It is important to note that on some 4D flow datasets, phase wrapping was observed, which was manually corrected within GTflow. The datasets affected with phase wrapping were the ones acquired with a spatial resolution of 0.5 mm^3^ and CS = 2.5–13, 1.0 mm^3^ and CS = 4.5 and 13, and 1.5 mm^3^ and CS = 6.5. Flow calculated in ROI C at the tube with ID = 2 mm was most affected, while ROI A and B were almost intact.

### 3.2. Velocity in an Aneurysm Model

**Figure 7:**
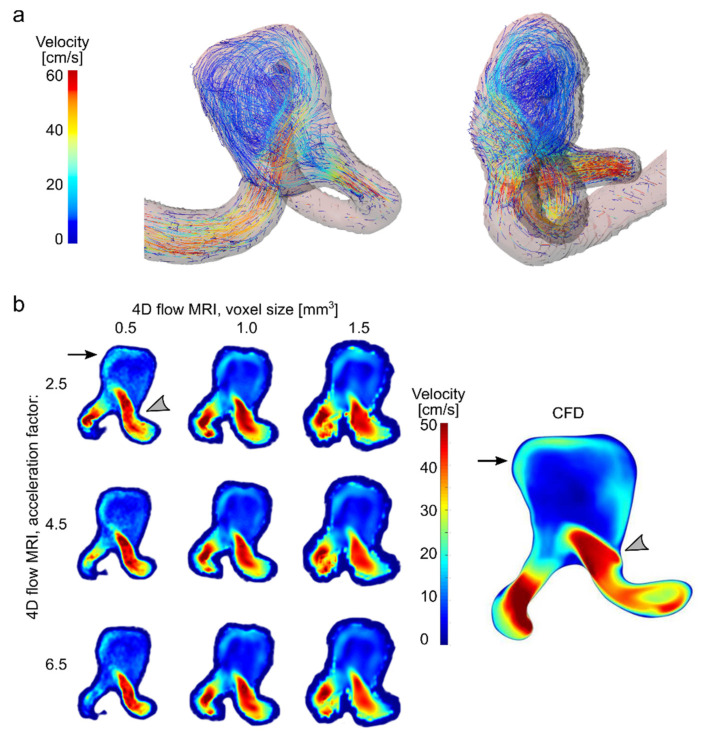
Effect of resolution and acceleration on velocity in an aneurysm flow model. (**a**) Visualization of velocity streamlines of the flow in a patient-derived aneurysm model, obtained by 4D flow MRI with an isotropic voxel size of 1 mm^3^ and a CS factor of 4.5. (**b**) Velocity field in patient-specific aneurysm model measured with 4D flow MRI and calculated with CFD. Note that a strong inflow jet from the parent artery into the aneurysm sac (gray asterisk) and residual flow on the opposite aneurysm wall (black arrows) were observed with all combinations of voxel sizes and CS factor of 4D flow MRI.

To give an overview of the flow pattern inside the aneurysm model, velocity streamlines were generated from 4D flow MRI data (Figure 7a). Blood enters in the distal part of the ostium, impinging on the aneurysm wall, while the velocity is continuously reduced until the blood reenters the parent vessel. The aneurysm has a regular shape, e.g., with no bulbs. Consequently, a single vortex forms, and a stagnation zone with very low-velocity values develops.

Flow in parental vessel and aneurysm was pulsatile at a time-averaged flow rate of 4.03 ± 1.34 and 2.99 ± 0.74 mL/s, respectively, as measured with 2D flow MRI (Figure S6). The flow was measured with 2D flow MRI experiments five times at parental vessel and aneurysm to test repeatability (Figure S7). The time-averaged repeatability coefficient was equal to 8.6 ± 4.1% and 6.6 ± 2.6% for parental vessel and aneurysm, respectively.

The peak velocity and stagnation zone were detected by CFD, too, and were reproduced qualitatively by all combinations of voxel sizes and CS acceleration factors of 4D flow MRI (Figure 7b).

**Figure 8:**
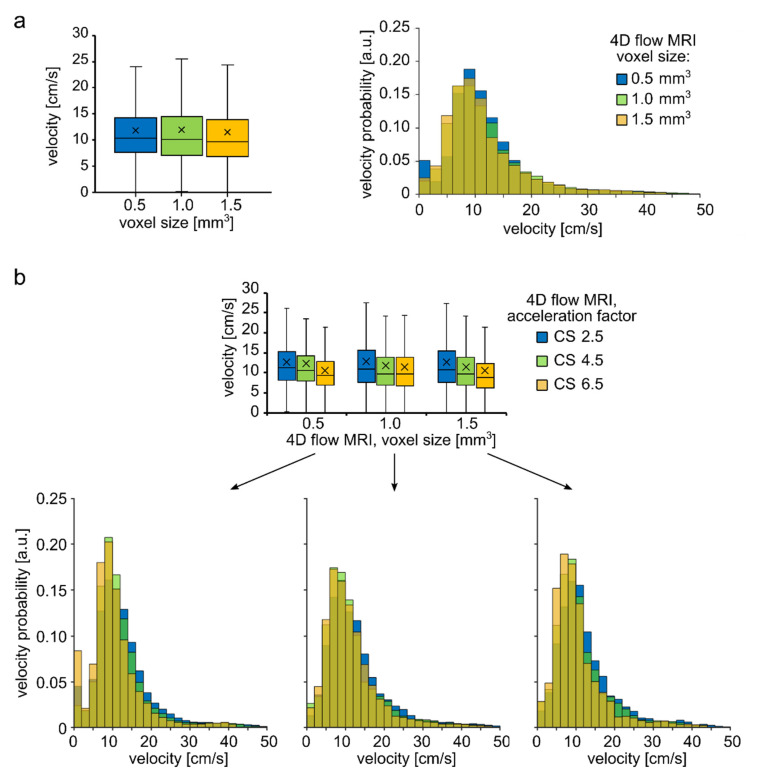
Quantitative analysis of velocity distribution within an aneurysm sac of the aneurysm model. (**a**) Boxplot and histogram of velocity magnitude distribution obtained with different voxel sizes regardless of the CS acceleration factor. (**b**) Histograms have a similar shape for the 4D flow MRI parameters considered. Moreover, the median velocity values were similar among the voxel sizes but were statistically different. Boxplot and histograms of velocity distribution were obtained with varying CS acceleration factors but with constant resolution. The trend of decreasing velocity with increasing CS acceleration factor was observed.

The medians of velocity distributions measured in 3D aneurysm ROI (Figure 1d left) with 4D flow MRI among three voxel sizes, regardless of CS factor, were found to be close to each other—10.4, 10.1, and 9.7 cm/s for a voxel size of 0.5, 1.0, and 1.5 mm^3^, respectively (Figure 8a, left). However, the velocity distributions were statistically different (Kruskal–Wallis test, *p*-value << 0.01, Figure 8a, right). The decreasing median velocity value was observed for an increasing acceleration factor while keeping the voxel size the same (Figure 8b). In particular, (1) a voxel size of 0.5 mm^3^ yielded a median velocity of 11.2, 10.5, and 9.4 cm/s for acceleration factors 2.5, 4.5, and 6.5, respectively; (2) a voxel size of 1.0 mm^3^ resulted in a median velocity of 10.9, 9.8, and 9.8 cm/s; and (3) a voxel size of 1.5 mm^3^ yielded a median velocity equal to 10.7, 9.7, and 8.9 cm/s. Note that the CS factor affected median values stronger than voxel size. For each spatial resolution, the velocity obtained with CS = 4.5 and 6.5 were compared against the one obtained with CS = 2.5. Statistical difference was observed for all cases (Wilcoxon rank-sum test, *p* << 0.01, Table S7). However, the changes did not exceed 11% for CS = 4.5 and 17% for CS = 6.5 in comparison to CS = 2.5 (Table S7). In addition, the median of velocity distribution obtained using 4D flow MRI with voxel size 0.5 mm^3^ and CS = 2.5 was compared to all other combinations of CS factors and voxel sizes. In this case, the difference between medians ranged from −20.82% (voxel size = 1.5 mm^3^, CS = 6.5) to −3.09 % (voxel size = 1 mm^3^, CS = 2.5). Overall, the median velocity obtained with voxel size 0.5 and CS = 2.5 was underestimated by average on −11.42 ± 6.1% in comparison to all other combinations of voxel size and CS factors used. 

## 4. Discussion

In previous studies, 4D flow MRI has been compared with numerous invasive and noninvasive methods [22,24,25,26,27]. However, the studies systematically addressing the impact of 4D flow MRI acquisition parameters on flow quantification are limited. In a recent review [41], the authors performed a systematic analysis on the comparability of the flow volume and peak velocity measured with 4D flow to 2D flow MRI in heart and great vessels. As a result, they found that 4D flow results are in the agreement with 2D flow in only 55% of the manuscript considered. The authors emphasize that 4D flow acquisition parameters have a great impact on flow accuracy, and each center needs to perform its quality assurance before using 4D flow MRI for clinical needs. In our work, we wanted to contribute to this awareness too. Thus, we aimed to assess the effect of spatial resolution and CS acceleration factors on flow quantification in small vessels. Firstly, a strong correlation (rho > 0.97) and linear relationship (R2 > 0.96) were observed between 2D and 4D flow MRI for all the various CS acceleration factors and spatial resolutions considered in the silicone tubes. This finding is in agreement with previously reported values. When comparing 4D flow with 2D flow MRI, transonic flow-sensor or Doppler US, a strong correlation was reported in assessing flow in a pulsatile phantom (rho = 0.96) [22], in healthy human volunteers (rho > 0.95) [42], in a swine aorta (rho = 0.86) [43], and in canine arteries (rho = 0.95 [28] and rho = 0.89 [24]).

### 4.1. Effect of Spatial Resolution and MR Acceleration on the Flow in Silicone Tubes

It is well known that the accuracy of phase-contrast imaging in small vessels is lower than in large ones because the relative number of voxels at the vessel’s boundaries increases with decreasing vessel diameter [44]. Overall, flow values between 2D and 4D flow MRI were close, and a linear slope ranged from 0.93 to 1.09 for the smaller voxel sizes of 0.5 and 1 mm^3^. However, flow was overestimated in comparison to 2D flow MRI for a greater voxel size (1.5 mm^3^), where the linear slope varied from 1.05 to 1.29. The flow was overestimated in all experiments by an average of 1.33 ± 8.31% and 0.24 ± 10.73% in comparison to 2D flow MRI and the US sensor, respectively, for all voxel sizes and CS acceleration factors used for 4D flow MRI. Flow was overestimated by 16%, as reported previously in a swine study (ID at different segments = 11.6, 16.55, and 18.1 mm, voxel size: 2.1 × 1.7 × 2.8 mm^3^, nROI = 5, 7, and 8) [43]. In another study, the flow was overestimated by 5% in a pulsatile phantom (ID = 20 mm) [22] compared to the transonic sensor. In some cases, the difference was eliminated when a high resolution was used (voxel size: 1.9 and 1.5 mm^3^ instead of 2.8 × 2.8 × 2.2 mm^3^, nROI = 10 and 13 instead of 7, respectively) [22]. However, another study reports that no statistical difference was found for flow estimated with 2D flow MRI obtained with 1 × 1 mm^2^ and 2 × 2 mm^2^ (single-slice acquisition) compared to the flow sensor (ID = 3.5 ± 0.7 mm, nROI = 2–4 instead of 1–2) [28]. The spatial resolution itself without a context on vessel diameters does not allow to estimate the flow quantification error. It might be no effect of spatial resolution seen because it was already sufficient resolution and its increase did not change anything, or it might not be sufficient in both cases, and the flow is calculated with error in both cases. In the recent review [41], the authors showed that the spatial resolution did not have a statistical impact on the agreement between 2D flow and 4D flow MRI; likely some other parameters had a stronger impact, or the spatial resolution was sufficient to eliminate an effect on the flow quantification error.

The flow estimation error is the greatest on the boundary voxels and, therefore, it depends on the number of boundary voxels $N_{boundary}≅\;\pi \cdot D/voxel\;size$ [44], where *D* corresponds to the diameter of the ROI. In other words, the error depends linearly on the number of voxels per vessel diameter (nROI). If this value is small, then it will limit the accuracy of the flow determination. We observed that in areas with less than six voxels per diameter, the RMS value decreases as the number of voxels increases. However, if more than seven voxels are used, the RMS value tends to plateau. The difference between median flow values obtained with 2D and 4D flow MRI (CS =2.5) was up to 20% for nROI in the range 2–5 voxels per diameter and less than 5% for nROI in the range 7–10 voxels per diameter. Previous studies have shown that an insufficient number of voxels per vessel diameter might lead to blood flow overestimation. Aristova et al. [45] investigated the accuracy of flow measurement with dual-venc 4D flow MRI accelerated with PEAK-GRAPPA (R = 2, 3, and 5) in comparison to 4D flow accelerated with GRAPPA (R = 2) using in vitro channels (ID = 4, 6, and 8 mm). In that study, flow measurement accuracy was 10–15% when nROI = 5, and less when more than five voxels were used, which is in accordance with our results. Another study reported simulated flow error to be less than 4% (nROI = 10), 20% (nROI = 4), and up to 60% (nROI = 2) [44]. In the same study, experimental MRI flow error was less than 9% (nROI = 10) and 20% (nROI = 5). In another study performed by Bouillot et al. [46], flow was overestimated by up to 120% (nROI = 1.3) and 60% (nROI = 2). However, in a different study, a weak trend of decreasing the flow error was apparent when more than four voxels per diameter were used [28]. Fewer voxels per diameter render flow determination less accurate. This effect must be considered when estimating the flow in small vessels. In addition to the MRI spatial resolution, it is desirable to report the size of the vessel diameter, too, or the number of voxels per diameter used for calculating flow.

Increasing spatial resolution might offer one solution for providing more voxels and better flow estimation. However, that cannot always be achieved easily and is compromised by longer measurement times and reduced signal-to-noise ratio (SNR). Using post-processing pipelines has been proposed as an alternative solution to decrease the error associated with boundary voxels. Recently, Boulleot et al. [46] suggested combining velocity information measured by 4D flow MRI with vessel geometry data measured with TOF or 3D rotational angiography to correct for insufficient spatial resolution. Globally, such correction led to 10–15% lower flow rates, and low sensitivity to the spatial resolution was observed when >2 voxels per diameter were used [46]. Another post-processing algorithm is based on the assumption that low flow at the boundaries induced small-phase shifts [47]. This algorithm reduced error from 36% without correction to less than 10% with correction. One more algorithm based on the predefined model-based method assumes a parabolic flow profile and cylindrical vessel geometry. It results in the reduction of errors from more than 50% to less than 10% (nROI > 5) [48]. Still, it is worth noting that even small uncertainties in flow definition can lead to a large variation in the determination of WSS when, for example, PC MRI flow is used to estimate aneurysm rupture risk with patient-specific CFD simulations [49].

Additionally, advanced accelerating imaging techniques can improve spatial resolution by reducing the examination time. Therefore, we also assessed the influence of the acceleration factor on flow measurements. No clear effect of the acceleration factor was found on the correlation coefficient, the linear slope. However, a trend of decreasing flow difference error and increasing RMS with increased CS factors was observed, although a small acceleration factor peak flow is better assessed for larger voxel sizes (1.0 and 1.5 mm^3^). Moreover, the smallest linear slope (1.05) was yielded when 1.5 mm^3^ voxels were used. Additionally, a higher acceleration factor (CS = 6.5) resulted in statistically higher RMS values than the ones obtained with a smaller acceleration factor (CS = 2.5); however, no statistical difference was found between CS = 2.5 and CS = 4.5. Additionally, the dependence of flow error from nROI was observed only for CS = 2.5 and 4.5; for CS = 6.5 and 13, some other factors likely play the dominant role. Previously, the feasibility of using acceleration factors of 4 to 10 [50] and even 10 to 30 [51] was demonstrated for in vivo aortic applications. Therefore, the use of higher CS factors might be appropriate. Yet, in the present study, an acceleration factor of 13 resulted in severe image distortions. No significant impact of CS = 13 on flow quantification was seen here likely due to the consistent placement of ROIs used for flow quantification. Still, peak flow was previously underestimated in the order of 25% [51] for an acceleration factor of 20, and 4% and 5% for acceleration factors of 8 and 13, respectively [42]. However, previously, Aristova et al. [45] showed no significant relationship between acceleration factor and flow measurement error for dual-venc 4D flow MRI accelerated with PEAK-GRAPPA. Overall, the number of studies considering the effect of acceleration of flow quantification even in the big vessels is limited. Moreover, it is worth noting that according to the review on 4D flow MRI in the heart and great vessels [41], most of the clinical centers do not use any acceleration technique at all. Recently, Pathrose et al. [52] studied CS 4D flow acquisitions in patients with aortic disease in comparison to 4D flow accelerated with GRAPPA. On average, the net flow was underestimated in the aortic arch by −7.1 ± 10.5%, −6.8 ± 13.8%, and −5.0 ± 10.7% for CS = 5.7, 7.7, and 10.2, respectively. However, at descending aorta, it was underestimated by −5.3 ± 11.8% only when CS = 10.2 was used. Moreover, at the ascending aorta, no significant impact of CS factor was found. Moreover, for some patients, the flow values were in good agreement between all 4D flow acquisition protocols. That being said, likely some other factors lead to the flow quantification error in combination with acceleration. In our study, on the contrary, flow overestimation was observed with increased CS factor. We hypothesize that the accelerated 4D flow acquisition is more capable of measuring more accurately peak flow values, and thus, better agreement between flow measured with CS = 6.5 and flow sensor was observed in Figure 2.

### 4.2. Velocity in an Aneurysm Model

As a neurovascular application of 4D flow MRI, we assessed the velocity field in the intracranial aneurysm model. Aneurysms may rupture, and a potentially fatal subarachnoid hemorrhage can develop [53]. Thus, aneurysms at risk of rupture need to be identified and treated. Some current options for treating aneurysms are based on flow disruption. Four-dimensional flow MRI has already been applied after aneurysm treatment to assess the blood flow changes [12,13]. Therefore, accurate assessment of aneurysm hemodynamics in vivo is desirable. Several studies compared 4D flow MRI velocity maps in the aneurysm to CFD and PIV [14,27,54]. However, studies addressing the impact of 4D flow MRI parameters on aneurysm flow are limited. In the present study, a good qualitative reproduction of time-averaged velocity magnitude maps calculated by CFD was achieved by all values of voxel sizes and acceleration factors. The main features of flow patterns, such as stagnation and high-velocity zones, were revealed with all voxel sizes and CS factors. Moreover, the velocity datasets measured with different voxel sizes yielded very close values of median velocity over the aneurysm sac. However, the larger voxel sizes resulted in smaller velocity values. This agrees with previous data by Roloff et al. [27], who reported underestimation of averaged velocity magnitude on 7.5 cm/s by 4D flow MRI (isotropic voxel size 0.57 mm^3^) compared to tomographic PIV (isotropic voxel size 0.23 mm^3^). However, when the PIV was downsampled to 4D flow MRI voxel size, the difference was reduced to 0.02 cm/s. Furthermore, further downsampling of the PIV data (isotropic voxel size 0.83 mm^3^) resulted in an average velocity magnitude reduction of 9.8 cm/s compared to the original data. The large voxel size likely represents a major factor in underestimating the velocity magnitude, which might be independent from the measurement technique. In another study, significant variation between velocity fields was observed in a comparison of 4D flow MRI (isotropic voxel size 0.5 mm^3^) and PIV (isotropic voxel size 0.17 mm^3^), which was eliminated after downsampling the spatial resolution of PIV [54].

The overall difference in velocity maps and median values acquired with various voxel sizes was relatively small in our study. Still, owing to the high-resolution secondary flow features, such as recirculation flow areas, can be captured, as discussed in Medero et al. [54]. Additionally, velocity-derived parameters such as WSS and OSI might also benefit from high resolution [55], as well as pressure [56]. The aneurysm considered in this study had a relatively simple shape, with no bulbs or aneurysm daughters. Therefore, only one vortex flow in the center of the aneurysm sac was present and well captured even by the coarse voxel size. The aneurysm investigated in our study was relatively large at 17.5 mm in the maximum dimension, resulting in 35, 18, and 12 voxels per maximum dimension using voxel sizes of 0.5, 1, and 1.5 mm^3^, respectively. Thus, spatial resolution was likely already sufficient for all voxel sizes. For smaller aneurysms with additional morphological features, a higher resolution is recommended.

The varying acceleration factor resulted in very close, but significantly different median velocity values for almost all cases. Additionally, the acceleration factor has a greater effect on velocity distribution than voxel size, likely owing to the relatively large aneurysm size. Moreover, the increased acceleration factor tended to decrease velocity. However, the changes did not exceed 11% for CS = 4.5 and 17% for CS = 6.5 in comparison to CS = 2.5, and no statistical difference was reported between the velocity field obtained with 4D flow MRI using another acceleration scheme [57]. The reduction in examination time gained by using a high acceleration factor can be used to improve spatial and temporal resolution. Here, we did not vary the temporal resolution; however, it may impact peak flow and velocity-derived parameters such as OSI [58,59].

### 4.3. Limitation

A limited number of models were considered in the present study: silicone tubes and one patient-specific aneurysm model. Other configurations of aneurysms or vessels might yield different results. Furthermore, this is an in vitro study only. It would be beneficial to vary spatial resolution and acceleration factor in vivo as well. In addition, some 4D flow MRI data were subjected to manual phase-wrapping correction, and some data could not be fully recovered, which may introduce errors in the flow estimation. The flow measured with 4D flow MRI data was compared to that measured with 2D flow MRI, representing another limitation of the study. Indeed, 2D flow MRI does not constitute a ground truth method, as it uses non-isotropic voxel sizes and non-real-time data collection (cine approach). Still, flow measured with 2D flow MRI was in good agreement with the flow values obtained with a flow sensor.

## 5. Conclusions

The spatial resolution affects the flow rate estimated by using 4D flow MRI. By coarse voxel size, flow is overestimated. The use of fewer than seven voxels per vessel diameter overestimated flow in more than 5% of flow measured with 2D flow MRI. Furthermore, we encourage reporting the inner diameter of the investigated vessel in addition to the spatial resolution of 4D flow MRI. This will make it possible to estimate the possible flow error. The velocity error in the aneurysm sac did not exceed 11% for CS = 4.5 in comparison to the velocity obtained using 4D flow MRI with CS = 2.5. Therefore, CS factors from 2.5–4.5 can be used to improve the spatial or temporal resolution for accurate quantification of flow rate and velocity.

## Figures and Tables

**Table 1:** Protocol parameters for the in-house 2D and 4D flow and TOF MRI sequences modified from the vendor protocol.

MRI Protocol	P1	P2	P3	P4	P5	P6	P7	P8
MRI sequence	2D flow	4D flow						TOF
model	tubes, aneurysm	tubes			aneurysm			aneurysm
TR/TE [ms]	9.4/6.2	10/6.3	7.5/4.6	6.6/4.0	10.6/6.4	7.2/4.4	6.5/3.9	25/5.8
vox. size [mm^3^]	0.5 × 0.5 × 4	0.5 × 0.5 × 0.5	1 × 1 × 1	1.5 × 1.5 × 1.5	0.5 × 0.5 × 0.5	1 × 1 × 1	1.5 × 1.5 × 1.5	0.25 × 0.40 × 050
FOV [mm^3^]	180 × 180	110 × 78 × 30			100 × 100 × 20			180 × 180 × 160
CS factor	2.5	2.5; 4.5; 6.5; 13			2.5; 4.5; 6.5			4.7
acq. Time [min]	2	11.2–57.5	2.7–14.2	1.2–6.2	28.5–73.2	7.4–18.8	3.2–8.2	20
card. Phase	24							-
Venc	60, 80	60			80			-

TR: repetition time; TE: echo time; FOV: field of view; vox. size: acquired voxel size; CS factor: acceleration factor of the compressed SENSE technique.

**Table 2:** Parameters of linear fit calculated for flow values acquired with 2D and 4D flow MRI data in silicone tubes. The linear slope was close to 1 for most 4D flow MRI parameters. The flow was overestimated (linear slope > 1.21) by 4D flow MRI acquired with 1.5 mm^3^ spatial resolution and accelerated by factors of 4.5 to 13.

Acquisition Parameters of 4D Flow MRI		A Linear Fit	
Spatial Resolution [mm^3^]	Acceleration Factor	Linear Slope	R^2^
0.5	2.5	1.08	0.99
	4.5	1.05	0.99
	6.5	1.07	0.97
	13	1.01	0.96
1.0	2.5	0.93	0.97
	4.5	1.06	0.97
	6.5	1.08	0.97
	13	1.09	0.97
1.5	2.5	1.05	0.97
	4.5	1.25	0.97
	6.5	1.21	0.97
	13	1.29	0.97

## Data Availability

In vitro 4D flow MRI data generated during this study is available at https://doi.org/10.5281/zenodo.4882572 (accessed on 3 February 2022). Digital geometry of patient-specific aneurysm model ready for 3D printing is available at https://doi.org/10.5281/zenodo.4423031 (accessed on 3 February 2022).

## Supplementary Materials

The following supporting information can be downloaded at: https://www.mdpi.com/article/10.3390/tomography8010038/s1, Figure S1: Time-resolved flow rate curves measured with US-sensor three times each 90 minutes. Slight variations are present in the curves indicating good temporal stability of the circulating system.; Figure S2: Scatter plots and Spearman-rank correlation coefficient between time-resolved net flow rate values obtained by 2D flow and US sensor. A correlation coefficient was 0.96.l; Figure S3: Scatter plots and Spearman-rank correlation coefficient between time-resolved net flow rate values obtained by 4D flow US sensor in silicone tubes. A strong correlation was observed for all voxel sizes and acceleration factors (rho > 0.94).; Figure S4: Box plots representing flow values obtained with 4D flow MRI and flow differences calculated between 4D flow MRI and US sensor.; Figure S5: Scatter and box plots for experiments performed on silicone tubes.; Figure S6: Time-resolved flow rate curves measured with 2D flow and 4D flow MRI in patient-specific aneurysm model.; Figure S7: Time-resolved flow rate curves measured with 2D flow MRI at ROI 1-3 (a top) of an aneurysm model.; Table S1: Results of Shapiro–Wilk test for normality of experimental data: flow rate values.; Table S2: Results of Shapiro–Wilk test for normality of experimental data: flow differences. Table S3: Summary statistics for flow rate values measured with US sensor, 2D and 4D flow MRI. Table S4: Summary statistics for flow difference calculated between 4D flow MRI and US sensor. Table S5: Summary statistics for flow difference calculated between 4D and 2D flow MRI. Table S6: Normalized RMS values calculated between flow rates obtained with 4D flow MRI and US sensor, 4D and 2D flow MRI. Table S7: Summary statistics for velocity distribution in aneurysm sac obtained with 4D flow MRI.

## References

[1] Zhao L., Barlinn K., Sharma V.K., Tsivgoulis G., Cava L.F., Vasdekis S.N., Teoh H.L., Triantafyllou N., Chan B.P., Sharma A. (2011). Velocity criteria for intracranial stenosis revisited. Stroke.

[2] Hope T.A., Hope M.D., Purcell D.D., von Morze C., Vigneron D.B., Alley M.T., Dillon W.P. (2010). Evaluation of intracranial stenoses and aneurysms with accelerated 4D flow. Magn. Reason. Imaging.

[3] Sforza D.M., Putman C.M., Cebral J.R. (2009). Hemodynamics of cerebral aneurysms. Annu. Rev. Fluid Mech..

[4] Meckel S., Stalder A.F., Santini F., Radü E.-W., Rüfenacht D.A., Markl M., Wetzel S.G. (2008). In vivo visualization and analysis of 3-D hemodynamics in cerebral aneurysms with flow-sensitized 4-D MR imaging at 3 T. Neuroradiology.

[5] Markl M., Wu C., Hurley M.C., Ansari S.A., Carroll T.J., Rahme R.J., Aoun S.G., Carr J., Batjer H., Bendok B.R. (2013). Cerebral arteriovenous malformation: Complex 3D hemodynamics and 3D blood flow alterations during staged embolization. J. Magn. Reson. Imaging.

[6] Markl M., Chan F.P., Alley M.T., Wedding K.L., Draney M.T., Elkins C.J., Parker D.W., Wicker R., Taylor C.A., Herfkens R.J. (2003). Time-resolved three-dimensional phase-contrast MRI. J. Magn. Reson. Imaging.

[7] Boussel L., Rayz V., Martin A., Acevedo-Bolton G., Lawton M.T., Higashida R., Higashida R., Smith W.S., Young W.L., Saloner D. (2009). Phase-contrast magnetic resonance imaging measurements in intracranial aneurysms in vivo of flow patterns, velocity fields, and wall shear stress: Comparison with computational fluid dynamics. Magn. Reson. Med..

[8] Van Ooij P., Potters W.V., Guédon A., Schneiders J.J., Marquering H.A., Majoie C.B., van Bavel E., Nederveen A.J. (2013). Wall shear stress estimated with phase contrast MRI in an in vitro and in vivo intracranial aneurysm. J. Magn. Reson. Imaging.

[9] Sotelo J., Urbina J., Valverde I., Tejos C., Irarrázaval P., Andia M.E., Uribe S., Hurtado D.E. (2016). 3D Quantification of wall shear stress and oscillatory shear index using a finite-element method in 3D CINE PC-MRI data of the thoracic aorta. IEEE Trans. Med. Imaging.

[10] Stalder A.F., Russe M.F., Frydrychowicz A., Bock J., Hennig J., Markl M. (2008). Quantitative 2D and 3D phase contrast MRI: Optimized analysis of blood flow and vessel wall parameters. Magn. Reson. Med..

[11] Morgan A.G., Thrippleton M.J., Wardlaw J.M., Marshall I. (2021). 4D flow MRI for non-invasive measurement of blood flow in the brain: A systematic review. J. Cereb. Blood Flow Metab..

[12] Brina O., Bouillot P., Reymond P., Luthman A.S., Santarosa C., Fahrat M., Lovblad K.O., Machi P., Delattre B.M.A., Pereira V.M. (2019). How Flow Reduction Influences the Intracranial Aneurysm Occlusion: A Prospective 4D Phase-Contrast MRI Study. Am J Neuroradiology..

[13] Pereira V.M., Brina O., Delattre B.M.A., Ouared R., Bouillot P., Erceg G., Schaller K., Lovblad K.O., Vargas M.I. (2015). Assessment of intra-aneurysmal flow modification after flow diverter stent placement with four-dimensional flow MRI: A feasibility study. J. NeuroInterventional Surg..

[14] Sindeev S., Arnold P.G., Frolov S., Prothmann S., Liepsch D., Balasso A., Berg P., Kaczmarz S., Kirschke J.S. (2018). Phase-contrast MRI versus numerical simulation to quantify hemodynamical changes in cerebral aneurysms after flow diverter treatment. PLoS ONE.

[15] Turski P., Scarano A., Hartman E., Clark Z., Schubert T., Rivera L., Wu Y., Wieben O., Johnson K. (2016). Neurovascular 4DFlow MRI (phase contrast MRA): Emerging clinical applications. Neurovascular Imaging.

[16] Schnell S., Wu C., Ansari S.A. (2016). 4D MRI flow examinations in cerebral and extracerebral vessels. Ready for clinical routine?. Curr. Opin. Neurol..

[17] Slesnick T.C., Hashemi S. (2018). 4-dimensional phase contrast imaging in congenital heart disease: How we do it. MAGNETOM Flash.

[18] Bollache E., Barker A.J., Dolan R.S., Carr J.C., van Ooij P., Ahmadian R., Powell A., Collins J.D., Geiger J., Markl M. (2018). k-t accelerated aortic 4D flow MRI in under two minutes: Feasibility and impact of resolution, k-space sampling patterns, and respiratory navigator gating on hemodynamic measurements. Magn. Reson. Med..

[19] Bollache E., Knott K.D., Jarvis K., Boubertakh R., Dolan R.S., Camaioni C., Collins L., Scully P., Rabin S., Treibel T. (2019). Two-Minute k-Space and Time–Accelerated Aortic Four-Dimensional Flow MRI: Dual-Center Study of Feasibility and Impact on Velocity and Wall Shear Stress Quantification. Radiol. Cardiothorac. Imaging.

[20] Giese D., Wong J., Greil G.F., Buehrer M., Schaeffter T., Kozerke S. (2014). Towards highly accelerated Cartesian time-resolved 3D flow cardiovascular magnetic resonance in the clinical setting. J. Cardiovasc. Magn. Reson..

[21] Hsiao A., Lustig M., Alley M.T., Murphy M., Chan F.P., Herfkens R.J., Vasanawala S.S. (2012). Rapid pediatric cardiac assessment of flow and ventricular volume with compressed sensing parallel imaging volumetric cine phase-contrast MRI. Am. J. Roentgenol..

[22] David A., Le Touze D., Warin-Fresse K., Paul-Gilloteaux P., Bonnefoy F., Idier J., Moussaoui S., Guerin P., Serfaty J.M. (2019). In-vitro validation of 4D flow MRI measurements with an experimental pulsatile flow model. Diagn. Interv. Imaging.

[23] Gabbour M., Rigsby C., Markl M., Schnell S., Jarvis K.B., de Freitas R.A., Popescu A.R., Robinson J.D. (2013). Comparison of 4D flow and 2D PC MRI blood flow quantification in children and young adults with congenital heart disease. J. Cardiovasc. Magn. Reson..

[24] Jiang J., Strother C., Johnson K., Baker S., Consigny D., Wieben O., Zagzebski J. (2011). Comparison of blood velocity measurements between ultrasound Doppler and accelerated phase-contrast MR angiography in small arteries with disturbed flow. Phys. Med. Biol..

[25] Medero R., Hoffman C., Roldán-Alzate A. (2018). Comparison of 4D flow MRI and particle image velocimetry using an in vitro carotid bifurcation model. Ann. Biomed. Eng..

[26] Berg P., Stucht D., Janiga G., Beuing O., Speck O., Thévenin D. (2014). Cerebral blood flow in a healthy circle of Willis and two intracranial aneurysms: Computational fluid dynamics versus four-dimensional phase-contrast magnetic resonance imaging. J. Biomech. Eng..

[27] Roloff C., Stucht D., Beuing O., Berg P. (2019). Comparison of intracranial aneurysm flow quantification techniques: Standard PIV vs stereoscopic PIV vs tomographic PIV vs phase-contrast MRI vs. CFD. J. Neurointerv. Surg..

[28] Hofman M.B.M., Visser F.C., Rossum A.C.V., Vink G.Q.M., Sprenger M., Westerhof N. (1995). In vivo validation of magnetic resonance blood volume flow measurements with limited spatial resolution in small vessels. Magn. Reson. Med..

[29] Wollschlaeger G., Wollschlaeger P.B., Lucas F.V., Lopez V.F. (1967). Experience and result with postmortem cerebral angiography performed as routine procedure of the autopsy. Am. J. Roentgenol..

[30] Zarrinkoob L., Ambarki K., Wåhlin A., Birgander R., Eklund A., Malm J. (2015). Blood flow distribution in cerebral arteries. J. Cereb. Blood Flow Metab..

[31] Pravdivtseva M.S., Peschke E., Lindner T., Wodarg F., Hensler J., Gabbert D., Voges I., Berg P., Barker A.J., Jansen O. (2021). 3D-printed, patient-specific intracranial aneurysm models: From clinical data to flow experiments with endovascular devices. Med Phys..

[32] Haselhoff E. (2017). Understanding How Compressed SENSE Makes MRI Faster.

[33] Geerts-Ossevoort L., de Weerdt E., Duijndam A., van IJperen G., Peeters H., Doneva M., Nijenhuis M., Huang A. (2018). Compressed SENSE. Speed Done Right. Every Time.

[34] Donoho D.L. (2006). Compressed sensing. IEEE Trans. Inf. Theory.

[35] Pruessmann K.P., Weiger M., Scheidegger M.B., Boesiger P. (1999). SENSE: Sensitivity encoding for fast MRI. Magnetic Resonance in Medicine.

[36] Pelc N.J., Bernstein M.A., Shimakawa A., Glover G.H. (1991). Encoding strategies for three-direction phase-contrast MR imaging of flow. J. Magn. Reson. Imaging.

[37] Khan M.A., Liu J., Tarumi T., Lawley J.S., Liu P., Zhu D.C., Lu H., Zhang R. (2017). Measurement of cerebral blood flow using phase contrast magnetic resonance imaging and duplex ultrasonography. J. Cereb. Blood Flow Metab..

[38] Schnell S., Ansari S.A., Vakil P., Wasielewski M., Carr M.L., Hurley M.C., Bendok B.R., Batjer H., Carroll T.J., Carr J. (2014). Three-dimensional hemodynamics in intracranial aneurysms: Influence of size and morphology. J. Magn. Reson. Imaging..

[39] Raunig D.L., McShane L.M., Pennello G., Gatsonis C., Carson P.L., Voyvodic J.T., Wahl R.L., Kurland B.F., Schwarz A.J., Gönen M. (2015). Quantitative imaging biomarkers: a review of statistical methods for technical performance assessment. Stat. Methods Med. Res..

[40] Shapiro-Wilk and Shapiro-Francia Normality Tests. https://www.mathworks.com/matlabcentral/fileexchange/13964-shapiro-wilk-and-shapiro-francia-normality-tests.

[41] Doyle C.M., Orr J., Greenwood J.P., Plein S., Tsoumpas C., Bissell M.M. (2021). Four-dimensional flow magnetic resonance imaging in the assessment of blood flow in the heart and great vessels: A systematic review. J. Magn. Reson. Imaging.

[42] Gottwald L.M., Peper E.S., Zhang Q., Coolen B.F., Strijkers G.J., Nederveen A.J., van Ooij P. Pseudo-Spiral Sampling and Compressed Sensing Reconstruction Provides Flexibility of Temporal Resolution in Accelerated Aortic 4D Flow MRI: A Comparison With k-t Principal Component Analysis. 2020, 33, e4255.

[43] Stam K., Chelu R.G., van der Velde N., van Duin R., Wielopolski P., Nieman K., Merkus D., Hirsch A. (2019). Validation of 4D flow CMR against simultaneous invasive hemodynamic measurements: A swine study. Int. J. Cardiovasc. Imaging.

[44] Wolf R.L., Ehman R.L., Riederer S.J., Rossman P.J. (1993). Analysis of systematic and random error in MR volumetric flow measurements. Magn. Reson. Med..

[45] Aristova M., Vali A., Ansari S.A., Shaibani A., Alden T.D., Hurley M.C., Jahromi B.S., Potts M.B., Markl M., Schnell S. (2019). Standardized evaluation of cerebral arteriovenous malformations using flow distribution network graphs AND dual- venc 4D Flow MRI. J. Magn. Reson. Imaging.

[46] Bouillot P., Delattre B.M.A., Brina O., Ouared R., Farhat M., Chnafa C., Steinman D.A., Lovblad K.O., Pereira V.M., Vargas M.I. (2018). 3D phase contrast MRI: Partial volume correction for robust blood flow quantification in small intracranial vessels. Magn. Reson. Med..

[47] Tang C., Blatter D.D., Parker D.L. (1995). Correction of partial-volume effects in phase-contrast flow measurements. J. Magn. Reson. Imaging.

[48] Hoogeveen R.M., Bakker C.J.G., Viergever M.A. (1999). MR phase-contrast flow measurement with limited spatial resolution in small vessels: Value of model-based image analysis. Magn. Reson. Med..

[49] Liu X., Kao E., Haraldsson H., Ballweber M., Martin A., Li Y., Wang Y., Saloner D. (2021). Identification of Intra-Individual Variation in Intracranial Arterial Flow by MRI and the Effect on Computed Hemodynamic Descriptors. MAGMA.

[50] Neuhaus E., Weiss K., Bastkowski R., Koopmann J., Maintz D., Giese D. (2019). Accelerated Aortic 4D Flow Cardiovascular Magnetic Resonance Using Compressed Sensing: Applicability, Validation and Clinical Integration. J. Cardiovasc. Magn. Reson..

[51] Peper E.S., Gottwald L.M., Zhang Q., Coolen B.F., van Ooij P., Nederveen A.J., Strijkers G.J. (2020). Highly accelerated 4D flow cardiovascular magnetic resonance using a pseudo-spiral Cartesian acquisition and compressed sensing reconstruction for carotid flow and wall shear stress. J. Cardiovasc. Magn. Reson..

[52] Pathrose A., Ma L., Berhane H., Scott M.B., Chow K., Forman C., Jin N., Serhal A., Avery R., Carr J. (2021). Highly accelerated aortic 4D flow MRI using compressed sensing: Performance at different acceleration factors in patients with aortic disease. Magn. Reson. Med..

[53] Brisman J.L., Song J.K., Newell D.W. (2006). Cerebral aneurysms. N. Engl. J. Med..

[54] Medero R., Ruedinger K., Rutkowski D., Johnson K., Roldán-Alzate A. (2020). In vitro assessment of flow variability in an intracranial aneurysm model using 4D flow MRI and tomographic PIV. Ann. Biomed. Eng..

[55] Brindise M.C., Rothenberger S., Dickerhoff B., Schnell S., Markl M., Saloner D., Rays V.L., Vlachos P.P. (2019). Multi-modality cerebral aneurysm haemodynamic analysis: In vivo 4D flow MRI, in vitro volumetric particle velocimetry and in silico computational fluid dynamics. J. R. Soc. Interface.

[56] Marlevi D., Schollenberger J., Aristova M., Ferdian E., Ma Y., Young A.A., Edelman E.R., Schnell S., Figueroa C.A., Nordsletten D.A. (2021). Noninvasive quantification of cerebrovascular pressure changes using 4D flow MRI. Magn. Reson. Med..

[57] Liu J., Koskas L., Faraji F., Kao E., Wang Y., Haraldsson H., Kefayati S., Zhu C., Ahn S., Laub G. (2018). Highly accelerated intracranial 4D flow MRI: Evaluation of healthy volunteers and patients with intracranial aneurysms. MAGMA.

[58] Cibis M., Potters W.V., Gijsen F.J., Marquering H., van Ooij P., van Bavel E., Wentzel J.J., Nederveen A.J. (2016). The effect of spatial and temporal resolution of cine phase contrast MRI on wall shear stress and oscillatory shear index assessment. PLoS ONE.

[59] Gottwald L.M., Töger J., Bloch K.M., Peper E.S., Coolen B.F., Strijkers G.J., van Ooij P., Nederveen A.J. (2020). High Spatiotemporal Resolution 4D Flow MRI of Intracranial Aneurysms at 7T in 10 Minutes. Am. J. Neuroradiol..

