# Supplementary material for: Influence of Spatial Resolution and Compressed SENSE Acceleration Factor on Flow Quantification with 4D Flow MRI at 3 Tesla
Source: Tomography. 2022 Feb 10;8(1):457–78. doi: 10.3390/tomography8010038 (PMC8880336; doi:10.3390/tomography8010038)
Supplement: Supplementary file 1 [file tomography-08-00038-s001.zip › tomography-1565298-supplementary.pdf]

Supporting information

Title: Influence of spatial resolution and compressed SENSE acceleration factor on flow quantification with 4D Flow MRI at 3 Tesla

Authors: Mariya S. Pravdivtseva [1*], Franziska Gaidzik [2], Philipp Berg [2], Patricia Ulloa[1], Naomi Larsen [3], Olav Jansen [3], Jan-Bernd Hövener and Mona Salehi Ravesh[1]

[1] Department of Radiology and Neuroradiology, University Medical Center Schleswig-Holstein (UKSH), Section Biomedical Imaging, Molecular Imaging North Competence Center (MOIN CC), Kiel University;

[2] Department of Fluid Dynamics and Technical Flows, Research Campus *STIMULATE*, Magdeburg University;

[3] Department of Radiology and Neuroradiology, University Medical Center Schleswig-Holstein (UKSH), Kiel University;

* Correspondence: mariya.pravdivtseva@rad.uni-kiel.de; Tel.: +49 (0) 431 500 16 533

Tables

2. Materials and Methods

2.5. Statistical analysis

Normality (Gaussian distribution) was tested for all variables (flow rate, flow difference) using the Shapiro–Wilk test (Table S1 and Table S2).

Table S1. Results of Shapiro–Wilk test for normality of experimental data: flow rate values.
The Shapiro-Wilk null hypothesis (h) is "data in X is sample from continuous normal distribution". h = 0, then do not reject the null hypothesis at significance level (p) equal to 0.05. h = 1, then reject the null hypothesis at significance level p.

method		tube ID [mm]							
		2		3		4		5	
		h	p	h	p	h	p	h	p
US sensor		1	< 0.01	1	< 0.01	1	< 0.01	1	< 0.01
2D flow MRI		1	0.01	1	0.01	1	< 0.01	1	0.01
4D flow MRI									
spatial resolution	CS	1		1		1	< 0.01	1	
[mm³]			0.01		0.01				0.01
0.5	2.5	1	0.01	1	0.01	1	< 0.01	1	0.01
	4.5	1	0.01	1	< 0.01	1	< 0.01	1	0.02
	6.5	1	< 0.01	1	0.01	1	< 0.01	1	0.01
	13	1	0.01	1	0.01	1	< 0.01	1	0.01
1	2.5	1	0.01	1	0.01	1	< 0.01	1	0.01
	4.5	1	0.01	1	0.01	1	< 0.01	1	0.01
	6.5	1	< 0.01	1	0.01	1	< 0.01	1	0.01
	13	1	0.01	1	0.01	1	< 0.01	1	0.01
1.5	2.5	1	0.01	1	0.01	1	< 0.01	1	0.01
	4.5	1	0.01	1	0.01	1	< 0.01	1	0.01
	6.5	1	0.01	1	0.01	1	< 0.01	1	0.01
	13	1	0.01	1	0.01	1	< 0.01	1	0.01

Table S2. Results of Shapiro–Wilk test for normality of experimental data: flow differences.
The Shapiro-Wilk null hypothesis (h) is "data in X is sample from continuous normal distribution". h = 0, then do not reject the null hypothesis at significance level (p) equal to 0.05. h = 1, then reject the null hypothesis at significance level p.

normalized flow difference between:	tube ID [mm]			
	2	3	4	5

		h	p	h	p	h	p	h	p
2D flow and US sensor		1	0.02	1	< 0.01	1	< 0.01	1	< 0.01
4D flow and US sensor									
vox. size [mm^3]	CS	1	0.02	1	0.01	0	0.34	1	0.04
0.5	2.5	0	0.24	1	0.01	1	0.03	1	0.02
	4.5	0	0.19	0	0.12	1	0.01	0	0.52
	6.5	1	0.02	1	< 0.01	1	< 0.01	0	0.13
	13	1	0.00	1	< 0.01	1	0.01	1	0.01
1	2.5	0	0.31	0	0.12	0	0.09	1	< 0.01
	4.5	0	0.14	0	0.08	1	0.01	0	0.19
	6.5	0	0.29	1	0.03	0	0.07	0	0.09
	13	1	0.01	1	< 0.01	0	0.07	1	< 0.01
1.5	2.5	0	0.09	0	0.14	0	0.20	0	0.81
	4.5	0	0.24	0	0.21	0	0.31	0	0.09
	6.5	0	0.23	1	0.01	0	0.19	0	0.06
	13	1	0.02	1	0.01	0	0.34	1	0.04
4D flow and 2D flow									
vox. size [mm^3]	CS								
0.5	2.5	1	< 0.01	1	< 0.01	1	< 0.01	1	< 0.01
	4.5	1	< 0.01	1	< 0.01	0	0.26	1	< 0.01
	6.5	0	0.09	1	< 0.01	0	0.06	1	0.01
	13	0	0.07	1	< 0.01	1	< 0.01	1	< 0.01
1	2.5	1	0.04	1	0.04	1	< 0.01	0	0.32
	4.5	1	0.05	1	0.00	0	0.90	1	0.01
	6.5	1	0.02	1	< 0.01	1	0.00	1	< 0.01
	13	0	0.10	1	0.01	0	0.48	1	< 0.01
1.5	2.5	0	0.26	0	0.05	0	0.14	1	0.04
	4.5	0	0.07	1	< 0.01	0	0.13	1	0.01
	6.5	1	0.01	1	< 0.01	1	< 0.01	1	< 0.01
	13	1	0.04	1	< 0.01	1	0.02	1	< 0.01

3. Results

3.1. Flow in silicone tubes

Table S3. Summary statistics for flow rate values measured with US sensor, 2D and 4D flow MRI. *A two-sided paired Wilcoxon signed rank test was conducted for the flow rate values measured with US sensor, 2D and 4D flow MRI in silicone tubes to assess the statistical difference between datasets at significance level p = 0.05. P values < 0.05 are highlighted in bold.*
Abbreviations: Q1 - lower quartile, Q3 - upper quartile, SD - standard deviation, nROI - voxel number per vessel diameter

**method**		**min**	**max**	**Q1**	**median**	**Q3**	**mean**	**SD**	**p value, MRI vs sensor**	**p value, 4D vs 2D flow**
**tube ID = 2 mm**										
US sensor		-0.01	1.81	0.12	0.23	0.91	0.55	0.60		
2D flow MRI		0.08	1.18	0.17	0.47	0.94	0.56	0.40	0.53	
4D flow MRI										
voxel [mm^3] / nROI	CS									
0.5 / 4.5	2.5	0.06	1.22	0.12	0.44	0.95	0.55	0.43	0.61	0.27
	4.5	0.05	1.20	0.14	0.43	0.90	0.53	0.41	0.86	0.24

	6.5	0.05	1.60	0.13	0.52	1.06	0.63	0.52	0.23	0.05
	13	0.05	1.22	0.12	0.44	0.98	0.54	0.42	0.65	**0.01**
1.0 / 2.2	2.5	0.16	1.14	0.24	0.54	0.96	0.59	0.37	0.23	0.08
	4.5	0.08	1.40	0.15	0.50	1.10	0.63	0.48	0.19	**0.02**
	6.5	0.04	1.40	0.13	0.49	1.08	0.62	0.50	0.17	**0.05**
	13	0.04	1.27	0.15	0.46	0.97	0.56	0.44	0.82	0.93
1.5 / 1.7	2.5	0.19	1.26	0.27	0.57	1.06	0.66	0.40	0.07	**< 0.01**
	4.5	0.09	1.50	0.17	0.55	1.18	0.68	0.53	0.07	**0.01**
	6.5	0.05	1.50	0.16	0.56	1.20	0.66	0.53	0.06	**0.01**
	13	0.05	1.41	0.14	0.50	1.09	0.61	0.49	0.20	0.09
**tube ID = 3 mm**										
US sensor		0.20	3.58	0.35	0.59	2.02	1.22	1.15		
2D flow MRI		0.29	2.52	0.33	0.75	1.81	1.06	0.80	0.46	
4D flow MRI										
voxel [mm^3] / nROI	CS									
0.5 / 6.5	2.5	0.21	2.67	0.27	0.68	1.72	1.03	0.87	0.06	0.24
	4.5	0.20	2.69	0.27	0.69	1.68	1.03	0.86	0.06	0.23
	6.5	0.23	3.18	0.28	0.79	2.00	1.20	1.02	0.67	**0.04**
	13	0.22	2.68	0.30	0.72	1.84	1.06	0.88	0.12	0.55
1.0 / 3.3	2.5	0.37	2.27	0.45	0.87	1.70	1.09	0.68	0.91	0.41
	4.5	0.23	2.90	0.31	0.76	1.88	1.13	0.94	0.36	0.32
	6.5	0.21	2.91	0.29	0.72	1.92	1.12	0.96	0.25	0.36
	13	0.21	2.65	0.28	0.66	1.76	1.03	0.87	**0.05**	0.19
1.5 / 2.3	2.5	0.43	2.44	0.52	0.97	1.87	1.18	0.72	0.51	**< 0.01**
	4.5	0.26	3.04	0.32	0.77	2.03	1.19	0.98	0.95	**0.02**
	6.5	0.20	2.99	0.28	0.79	1.99	1.16	0.99	0.30	0.57
	13	0.19	2.94	0.25	0.74	1.98	1.13	0.99	0.21	0.55
**tube ID = 4 mm**										
US sensor		0.69	6.26	0.82	1.30	3.61	2.31	1.90		
2D flow MRI		0.54	4.48	0.78	1.67	3.37	2.11	1.41	0.35	
4D flow MRI										
voxel [mm^3] / nROI	CS									
0.5 / 8.1	2.5	0.41	4.93	0.64	1.66	3.55	2.13	1.60	0.17	0.89
	4.5	0.41	4.86	0.59	1.59	3.35	2.01	1.52	0.06	0.18
	6.5	0.35	4.04	0.48	1.14	2.65	1.63	1.29	**< 0.01**	**< 0.01**
	13	0.33	4.44	0.61	1.50	3.12	1.91	1.44	**0.01**	0.07
1.0 / 4.0	2.5	0.74	4.27	1.00	1.93	3.47	2.20	1.29	0.73	0.04
	4.5	0.44	5.14	0.63	1.68	3.61	2.15	1.61	0.28	0.61
	6.5	0.36	4.49	0.55	1.44	3.06	1.81	1.40	**< 0.01**	**< 0.01**
	13	0.39	4.73	0.57	1.61	3.44	2.01	1.53	0.06	0.09
1.5 / 2.9	2.5	0.87	4.77	1.14	2.12	3.83	2.50	1.43	0.10	**< 0.01**
	4.5	0.46	5.55	0.74	1.89	4.01	2.43	1.80	0.23	**0.03**
	6.5	0.39	5.09	0.63	1.61	3.46	2.13	1.65	0.19	0.86
	13	0.34	5.63	0.65	1.87	3.99	2.37	1.86	0.93	0.06
**tube ID = 5 mm**										
US sensor		1.11	7.94	1.19	1.83	4.80	3.07	2.35		
2D flow MRI		0.85	5.66	1.12	2.07	4.04	2.64	1.69	**0.03**	
4D flow MRI										
voxel [mm^3] / nROI	CS									
0.5 / 9.6	2.5	0.66	6.23	0.95	2.03	4.27	2.64	1.90	**0.01**	0.65
	4.5	0.68	6.09	0.88	2.05	4.23	2.62	1.87	**0.02**	0.61
	6.5	0.67	6.93	1.01	2.28	4.68	2.91	2.10	0.36	**0.03**

	13	0.70	6.04	0.92	1.94	4.12	2.61	1.87	**0.06**	0.39
1.0 / 4.8	2.5	1.00	5.33	1.34	2.34	4.04	2.71	1.51	0.42	0.49
	4.5	0.63	5.98	0.91	1.92	4.12	2.58	1.86	**< 0.01**	0.15
	6.5	0.71	6.62	0.99	2.21	4.57	2.84	2.06	0.19	0.12
	13	0.73	6.58	0.94	2.12	4.50	2.74	2.01	**0.03**	0.84
1.5 / 3.3	2.5	1.22	5.97	1.55	2.56	4.48	3.07	1.67	0.33	**< 0.01**
	4.5	0.87	7.39	1.15	2.39	4.93	3.16	2.24	0.38	**< 0.01**
	6.5	0.75	7.45	0.98	2.49	4.93	3.12	2.29	0.82	**0.03**
	13	0.82	7.69	1.04	2.53	5.05	3.21	2.35	0.91	0.01

Table S4. Summary statistics for flow difference calculated between 4D flow MRI and US sensor. *A two-sided paired Wilcoxon signed rank test was conducted to compare flow differences with +5, -5, +10 and -10 % at significance level p = 0.05. P values < 0.05 are highlighted in bold. Only datasets which were different in Table S3 were evaluated here. Abbreviations: Q1 - lower quartile, Q3 - upper quartile, SD - standard deviation, nROI - voxel number per vessel diameter*

**method**		**min**	**max**	**Q1**	**median**	**Q3**	**mean**	**SD**	**p value**	**compare to**
**tube ID = 2 mm**										
4D flow MRI										
voxel [mm^3] / nROI	CS									
0.5 / 4.5	2.5	-115.56	57.65	-22.00	8.20	33.85	-1.29	48.73		
	4.5	-113.63	66.62	-26.69	0.69	37.84	-4.91	50.75		
	6.5	-56.42	88.77	-19.29	9.58	44.93	14.66	42.46		
	13	-126.90	54.13	-20.86	4.55	30.14	-2.88	48.08		
1.0 / 2.2	2.5	-125.09	68.53	-2.76	27.03	47.88	7.51	57.03		
	4.5	-75.31	82.92	-16.54	11.76	49.87	13.50	46.40		
	6.5	-87.26	74.18	-19.23	17.45	53.20	11.73	44.23		
	13	-96.61	67.76	-24.75	4.69	36.39	1.08	47.07		
1.5 / 1.7	2.5	-101.01	78.20	1.14	30.24	59.25	19.18	51.83		
	4.5	-55.34	101.92	-14.36	12.71	64.53	22.93	48.53		
	6.5	-59.56	81.11	-18.23	19.70	56.14	19.76	43.07		
	13	-78.03	71.09	-21.44	14.30	43.48	10.34	42.07		
**tube ID = 3 mm**										
4D flow MRI										
voxel [mm^3] / nROI	CS									
0.5 / 6.5	2.5	-100.14	34.79	-26.41	-6.56	2.71	-15.12	35.49		
	4.5	-101.45	32.41	-25.11	-6.15	1.93	-15.31	35.91		
	6.5	-76.98	49.58	-11.49	0.66	8.35	-1.92	30.82		
	13	-82.97	20.79	-17.97	-8.08	8.72	-12.77	29.22		
1.0 / 3.3	2.5	-109.92	32.00	-28.06	4.14	17.32	-10.92	42.15		
	4.5	-85.05	47.09	-18.86	-2.61	5.71	-7.25	33.45		
	6.5	-75.07	34.03	-15.26	-6.22	5.71	-8.17	28.62		
	13	-102.16	41.30	-30.62	-9.09	2.15	-15.75	37.21	0.32	-5
1.5 / 2.3	2.5	-100.65	34.44	-15.88	10.25	28.28	-2.97	40.65		
	4.5	-72.33	44.06	-10.47	-0.25	10.96	-2.62	28.22		
	6.5	-86.44	69.68	-20.30	-5.53	5.91	-5.11	39.48		
	13	-65.44	14.29	-14.85	-3.48	7.34	-7.72	21.14		
**tube ID = 4 mm**										
4D flow MRI										

voxel [mm³] / nROI	CS									
0.5 / 8.1	2.5	-82.83	69.04	-23.31	-8.60	4.57	-7.65	37.85		
	4.5	-75.48	22.93	-19.82	-8.90	6.33	-12.90	26.41		
	6.5	-114.54	22.62	-47.63	-19.08	-10.38	-29.54	37.92	**0.02**	**-10**
	13	-81.31	9.81	-21.65	-11.89	5.07	-17.13	26.73	0.68	-10
1.0 / 4.0	2.5	-88.99	39.27	-11.98	3.34	17.57	-4.71	35.10		
	4.5	-67.69	30.45	-16.79	-4.26	10.51	-6.93	25.62		
	6.5	-94.43	16.84	-28.66	-13.92	-2.60	-21.51	30.58	0.17	-10
	13	-76.56	29.24	-19.73	-9.29	4.60	-12.77	27.95		
1.5 / 2.9	2.5	-68.19	65.22	0.31	12.92	25.00	8.26	34.43		
	4.5	-37.08	37.77	-12.54	7.72	24.40	5.39	21.45		
	6.5	-71.02	53.22	-19.65	-7.76	3.53	-7.64	31.74		
	13	-46.80	67.12	-17.80	-3.48	12.51	2.52	29.84		
**tube ID = 5 mm**										
4D flow MRI										
voxel [mm³] / nROI	CS									
0.5 / 9.6	2.5	-75.61	23.32	-22.42	-9.53	-0.24	-13.95	25.20	0.16	-5
	4.5	-78.48	22.03	-21.89	-11.21	0.34	-14.71	25.80	0.12	-5
	6.5	-60.55	34.76	-16.80	-2.83	7.83	-5.09	23.09		
	13	-92.22	51.25	-29.82	-12.92	-5.50	-14.86	36.62		
1.0 / 4.8	2.5	-97.82	38.16	-21.87	-2.50	8.16	-11.70	37.19		
	4.5	-75.71	9.19	-19.97	-8.95	1.02	-15.75	23.05	0.09	-5
	6.5	-62.11	26.91	-15.60	-5.41	5.24	-7.52	21.89		
	13	-72.89	34.43	-15.95	-8.61	-0.96	-10.54	26.08	0.15	-5
1.5 / 3.3	2.5	-70.10	27.49	-5.00	8.34	19.89	-0.03	27.91		
	4.5	-40.03	35.27	-10.15	2.98	16.15	3.02	18.97		
	6.5	-55.07	66.50	-13.85	-1.82	9.84	1.88	29.97		
	13	-49.08	70.81	-12.14	-1.02	14.99	4.73	30.10		

Table S5. Summary statistics for flow difference calculated between 4D and 2D flow MRI. *A two-sided paired Wilcoxon signed rank test was conducted to compare flow differences with +5, -5, +10 and -10 % at significance level p = 0.05. P values < 0.05 are highlighted in bold. Only datasets which were different in Table S3 were evaluated here.*
Abbreviations: Q1 - lower quartile, Q3 - upper quartile, SD - standard deviation, nROI - voxel number per vessel diameter

**method**		**min**	**max**	**Q1**	**median**	**Q3**	**mean**	**SD**	**p value**	**compare to**
**tube ID = 2 mm**										
4D flow MRI										
voxel [mm³] / nROI	CS									
0.5 / 4.5	2.5	-12.69	16.19	-9.65	-5.69	3.91	-2.26	9.44		
	4.5	-38.42	8.89	-15.90	-0.26	3.90	-5.84	14.08		
	6.5	-23.00	75.62	-6.65	5.99	32.83	13.53	27.94	0.21	5

	13	-12.74	12.89	-9.11	-5.98	-0.14	-3.83	6.92	0.73	-5
1.0 / 2.2	2.5	-11.15	36.32	-7.72	4.28	16.43	6.45	14.99		
	4.5	-19.36	42.53	-1.15	7.45	34.63	12.38	20.93	0.15	5
	6.5	-11.13	44.97	-3.71	5.41	27.54	10.64	18.40	0.29	5
	13	-27.19	20.37	-12.03	-0.41	13.74	0.08	14.80		
1.5 / 1.7	2.5	13.99	21.32	16.41	18.31	20.20	18.01	2.24		
	4.5	-27.00	66.72	-1.12	14.53	55.46	21.72	31.19		
	6.5	-11.77	57.22	-1.71	11.84	42.87	18.59	24.53	0.18	10
	13	-17.86	41.48	-3.60	2.95	24.71	9.26	18.17		
**tube ID = 3 mm**										
4D flow MRI										
voxel [mm³] / nROI	CS									
0.5 / 6.5	2.5	-13.95	18.08	-10.87	-6.00	2.95	-2.73	10.71		
	4.5	-14.23	18.83	-11.75	-5.27	3.30	-2.94	10.65		
	6.5	-8.29	61.72	-5.52	4.29	23.34	12.40	21.87	0.33	5
	13	-9.12	15.40	-5.83	-1.46	2.72	-0.04	7.75		
1.0 / 3.3	2.5	-27.23	20.76	-6.46	5.99	13.85	2.09	14.85		
	4.5	-10.89	39.67	-6.50	-0.87	18.02	6.29	16.40		
	6.5	-9.55	36.31	-7.75	-1.35	15.37	5.24	15.48		
	13	-19.74	23.72	-13.74	-6.47	3.35	-3.45	13.36		
1.5 / 2.3	2.5	-8.63	23.55	7.44	12.14	18.50	11.20	9.52	0.29	10
	4.5	-5.68	49.28	-2.40	3.38	25.27	11.60	17.58	0.28	5
	6.5	-15.90	59.95	-10.66	-2.95	22.32	8.75	25.30		
	13	-13.94	45.20	-7.62	-5.58	22.74	5.76	19.01		
**tube ID = 4 mm**										
4D flow MRI										
voxel [mm³] / nROI	CS									
0.5 / 8.1	2.5	-8.96	22.65	-7.94	-4.61	11.53	1.04	10.91		
	4.5	-34.45	17.65	-9.87	-2.85	3.42	-4.70	13.78		
	6.5	-34.78	-8.99	-31.29	-23.75	-15.17	-22.90	8.82	**< 0.01**	**-10**
	13	-52.11	8.76	-19.18	-2.59	3.96	-9.32	17.59		
1.0 / 4.0	2.5	-16.87	13.18	-0.91	9.56	10.51	4.26	9.66	0.65	5
	4.5	-27.06	30.88	-8.44	1.20	15.07	1.83	15.75		
	6.5	-41.11	0.25	-14.70	-10.67	-9.01	-14.12	9.68	0.1	-10
	13	-27.55	12.62	-10.00	-5.85	4.14	-4.55	11.22		
1.5 / 2.9	2.5	11.82	26.27	15.48	17.07	21.28	18.45	4.31	**< 0.01**	**10**
	4.5	-25.65	57.80	-4.27	10.74	40.24	15.31	26.99	0.36	10
	6.5	-12.97	28.75	-6.95	-3.95	8.16	1.05	11.81		
	13	-14.38	54.41	-6.74	5.88	27.22	12.17	21.98		
**tube ID = 5 mm**										
4D flow MRI										
voxel [mm³] / nROI	CS									
0.5 / 9.6	2.5	-10.40	23.05	-7.86	-6.16	7.91	0.16	11.39		
	4.5	-9.41	19.45	-8.41	-5.90	6.17	-0.73	10.06		
	6.5	-7.14	48.34	-4.14	4.22	20.85	10.47	17.09	0.38	5
	13	-21.80	51.00	-17.06	-10.18	9.34	-0.91	23.22		
1.0 / 4.8	2.5	-23.46	33.21	-8.10	3.56	6.96	2.78	15.32		
	4.5	-9.43	12.81	-7.58	-4.19	2.56	-1.94	6.86		
	6.5	-7.78	36.41	-5.20	0.95	20.00	7.64	15.09		
	13	-12.27	41.24	-7.11	-4.36	13.03	4.12	16.61		
1.5 / 3.3	2.5	11.43	20.44	13.30	16.57	19.20	16.36	3.04	**< 0.01**	**10**
	4.5	-1.93	65.80	1.69	13.35	33.79	19.91	21.22	0.12	10
	6.5	-6.18	80.11	-2.99	5.18	35.30	18.58	28.12	0.61	10

	13	-5.57	88.95	-0.74	6.36	38.68	21.90	30.23	0.30	10

Table S6. Normalized RMS values calculated between flow rates obtained with 4D flow MRI and US sensor, 4D and 2D flow MRI.

**method**		**4D flow MRI vs US sensor**				**4D flow MRI vs 2D flow MRI**			
		**tube ID [mm]**				**tube ID [mm]**			
		2	3	4	5	2	3	4	5
4D flow MRI									
voxel [mm³]	CS								
0.5	2.5	0.48	0.38	0.38	0.28	0.10	0.11	0.11	0.11
	4.5	0.50	0.38	0.29	0.29	0.15	0.11	0.14	0.10
	6.5	0.44	0.30	0.47	0.23	0.31	0.25	0.24	0.20
	13	0.47	0.31	0.31	0.39	0.08	0.08	0.20	0.23
1	2.5	0.56	0.43	0.35	0.38	0.16	0.15	0.10	0.15
	4.5	0.47	0.34	0.26	0.28	0.24	0.17	0.16	0.07
	6.5	0.45	0.29	0.37	0.23	0.21	0.16	0.17	0.17
	13	0.46	0.40	0.30	0.28	0.14	0.14	0.12	0.17
1.5	2.5	0.54	0.40	0.35	0.27	0.18	0.15	0.19	0.17
	4.5	0.53	0.28	0.22	0.19	0.37	0.21	0.31	0.29
	6.5	0.47	0.39	0.32	0.29	0.30	0.26	0.12	0.33
	13	0.42	0.22	0.29	0.30	0.20	0.19	0.25	0.37

3.2. *Velocity in an aneurysm model*

Table S7. **Summary statistics for velocity distribution in aneurysm sac obtained with 4D flow MRI.** *A two-sided Wilcoxon rank sum test was conducted to compare velocity distribution obtained using 4D flow MRI with CS 4.5 and 6.5 against CS 2.5 for each voxel size.*
Abbreviations: Q1 - lower quartile, Q3 - upper quartile, SD - standard deviation, nROI - voxel number per vessel diameter

**method**		**min**	**max**	**Q1**	**median**	**Q3**	**mean**	**SD**	**difference between medians [%]**	**p value**
4D flow MRI										
voxel [mm³]	CS									
0.5	2.5	0.25	53.86	8.08	11.23	15.30	12.72	7.76		
	4.5	0.07	88.71	8.03	10.52	14.19	12.25	7.49	-6.38	<< 0.01
	6.5	0.01	48.14	6.99	9.38	12.78	10.54	6.82	-16.50	<< 0.01
1.0	2.5	0.11	54.71	7.60	10.89	15.57	12.89	8.19		
	4.5	0.08	77.48	6.93	9.79	13.83	11.71	7.62	-10.09	<< 0.01
	6.5	0.11	49.23	6.72	9.75	13.81	11.44	7.30	-10.39	<< 0.01
1.5	2.5	0.10	50.84	7.60	10.70	15.46	12.60	7.84		
	4.5	0.10	46.53	6.93	9.69	13.84	11.38	7.12	-9.46	<< 0.01
	6.5	0.05	51.98	6.25	8.89	12.35	10.53	6.97	-16.87	<< 0.01

Figures

2. Materials and Methods

2.1. Flow models and circulation setup

Stability of the flow setup used for the MRI experiment with silicone tubes were tested over three hours (Figure S1). Straight tube with ID = 4 mm was connected to a pulsatile pump (PD-1100, BDC Laboratories, USA). The flow was measured with flow sensor every 90 minutes. Slight variations in flow profile are visible, however, the measured flow was very similar, mean flow values were 4.2 ± 1.7 ml/s (0 minutes), 4.5 ± 1.78 ml/s (90 minutes) and 4.3 ± 1.8 ml/s (150 minutes).

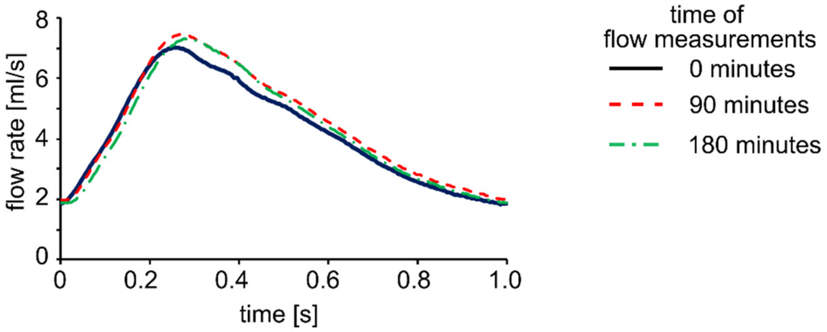

Figure S1. Time-resolved flow rate curves measured with US-sensor three times each 90 minutes. Slight variations are present in the curves indicating good temporal stability of the circulating system.

3. Results

3.1. Flow in silicone tubes

Flow measured with US sensor and with 2D flow MRI was strongly correlated (rho = 0.96, Figure S2). In addition, a linear fit was performed on US sensor and 2D flow MRI flow values ($R^2 > 0.92$). The linear slope was equal to 1.27.

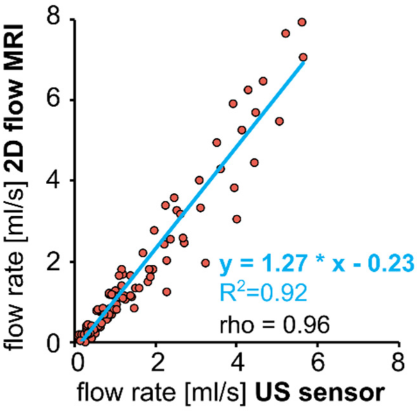

Figure S2. Scatter plots and Spearman-rank correlation coefficient between time-resolved net flow rate values obtained by 2D flow and US sensor. A correlation coefficient was 0.96.

A strong correlation (rho > 0.94) was observed between US sensor and 4D flow MRI for all voxel sizes and CS acceleration factors (Figure S3).

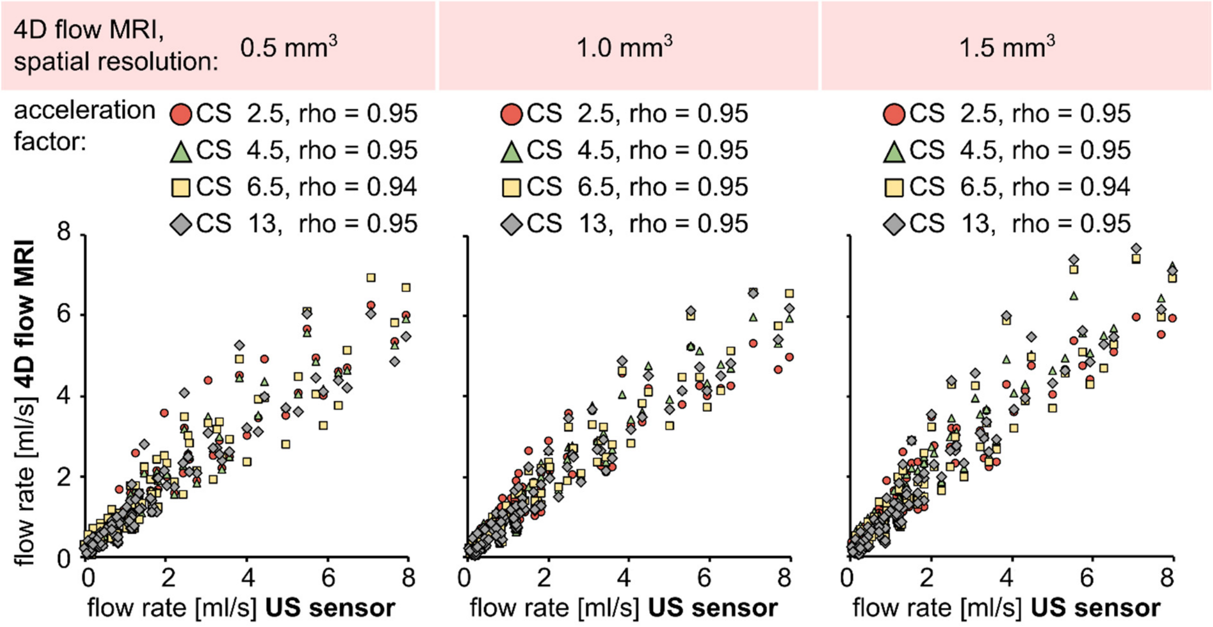

Figure S3. Scatter plots and Spearman-rank correlation coefficient between time-resolved net flow rate values obtained by 4D flow US sensor in silicone tubes. A strong correlation was observed for all voxel sizes and acceleration factors (rho > 0.94).

Overall, the flow was overestimated in all experiments, by an average of 1.33 ± 8.31 % for all voxel sizes and CS acceleration factors used. A two-sided paired Wilcoxon signed-rank test revealed the statistical difference between flow values measured with 4D flow MRI and US sensor for some combinations of spatial resolution and CS factors (Figure S4 left).

Flow difference between 4D flow MRI ranged from –19.08 (tube ID = 4 mm, voxel size = 0.5 mm3, CS = 6.5) to +30.24 % (tube ID = 2 mm, voxel size = 1.5 mm3, CS = 2.5). However, the median of flow difference calculated between 4D and US sensor was statistically higher than 10 % only voxel size of 1.5 mm3 and CS = 6.5 for tube ID = 4 (p < 0.001, Figure S4 right).

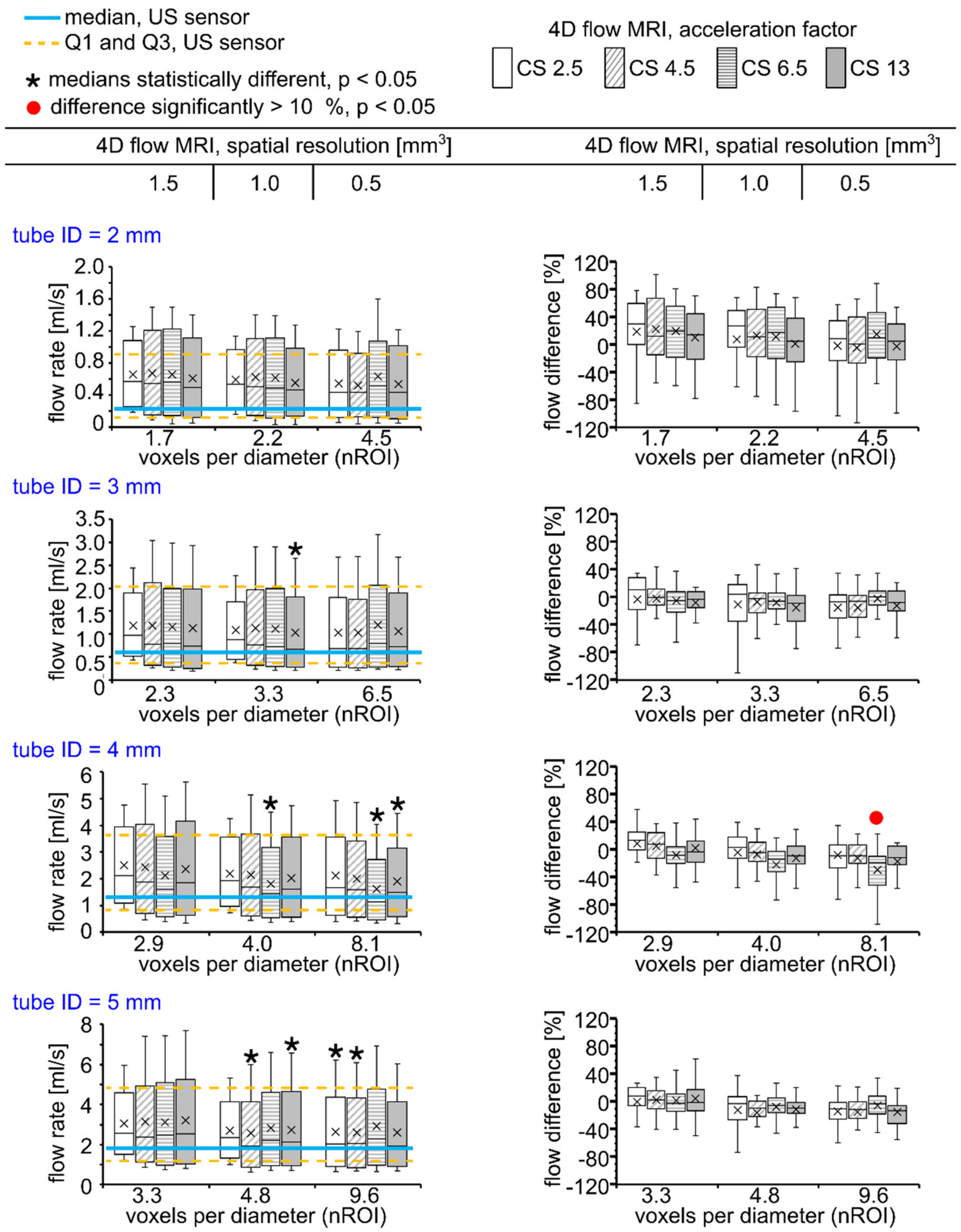

Figure S4. Box plots representing flow values obtained with 4D flow MRI (left) and flow differences calculated between 4D flow MRI and US sensor (right). Mostly the use of voxel size of 1.5 mm3 resulted in statistically different flow median values ($p < 0.001$, star sign, left, a two-sided paired Wilcoxon signed-rank test). However, the difference was significantly higher than 10 % only for one dataset ($p < 0.001$, red circle, right, a two-sided paired Wilcoxon signed-rank test).

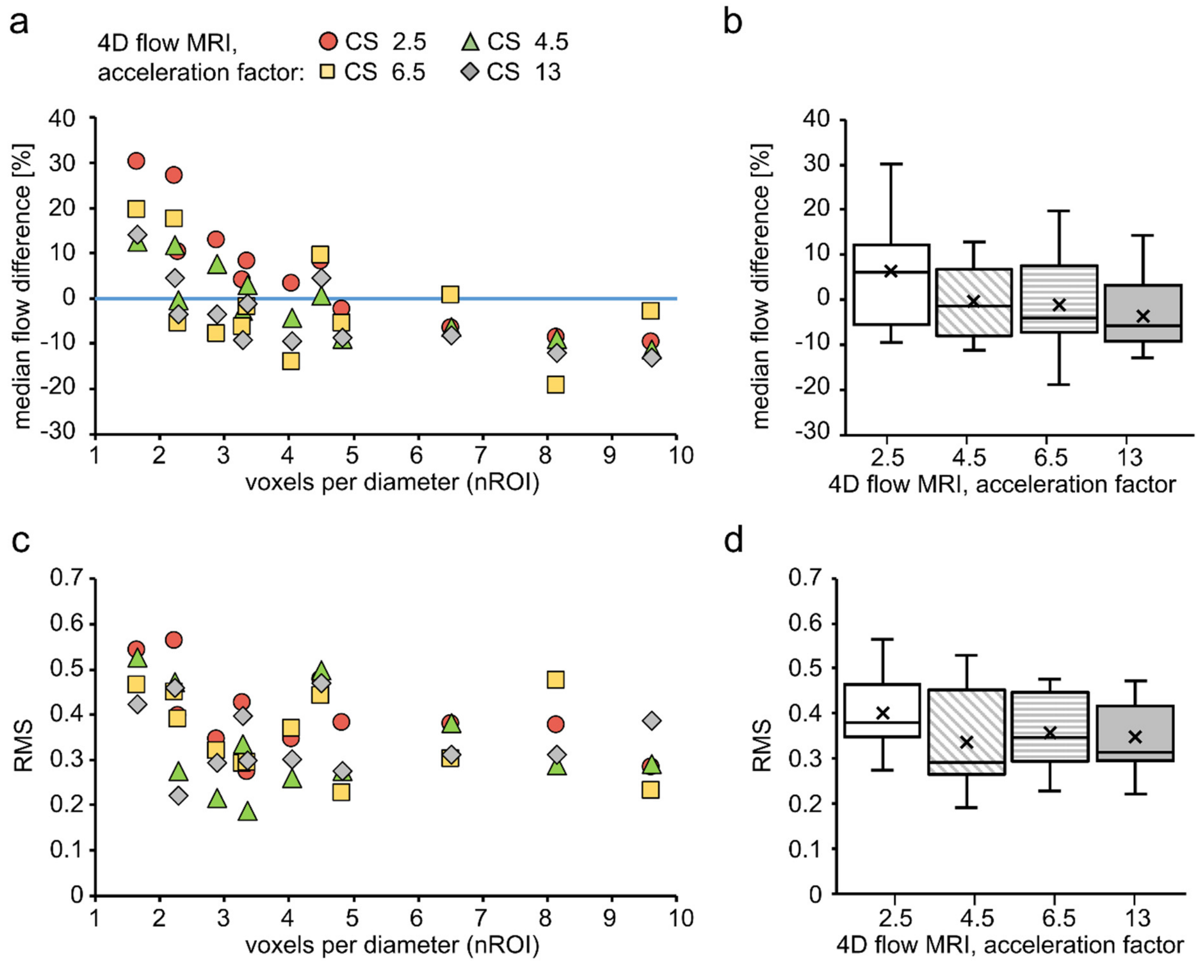

Figure S5. Scatter (left) and box plots (right) for experiments performed on silicone tubes. (a) The median difference between flow rate values measured with 4D flow MRI and US sensor tended to decrease with increasing voxel number per diameter. (b) Median flow difference tended to decrease with increased CS factor (c) RMS tended to decrease with increasing voxel number per diameter. (d) RMS tended to decreased with increased CS factor. For creating box plots in (b, d), RMS and flow difference values obtained by 4D flow MRI with various nROIs but with the same CS factor were joined together to make one dataset. Therefore, the dependency of data on nROI was excluded.

3.2. *Velocity in an aneurysm model*

The aneurysm was supplied with a pulsitile flow at time-averaged flow rate of 3.8 ml/s as measured with US sensor. Flow in parental vessel and aneurysm was pulsatile at time-averaged flow rate of 4.03 ± 1.34 and 2.99 ± 0.74 ml/s, respectively, as measured with 2D flow MRI (Figure S6).

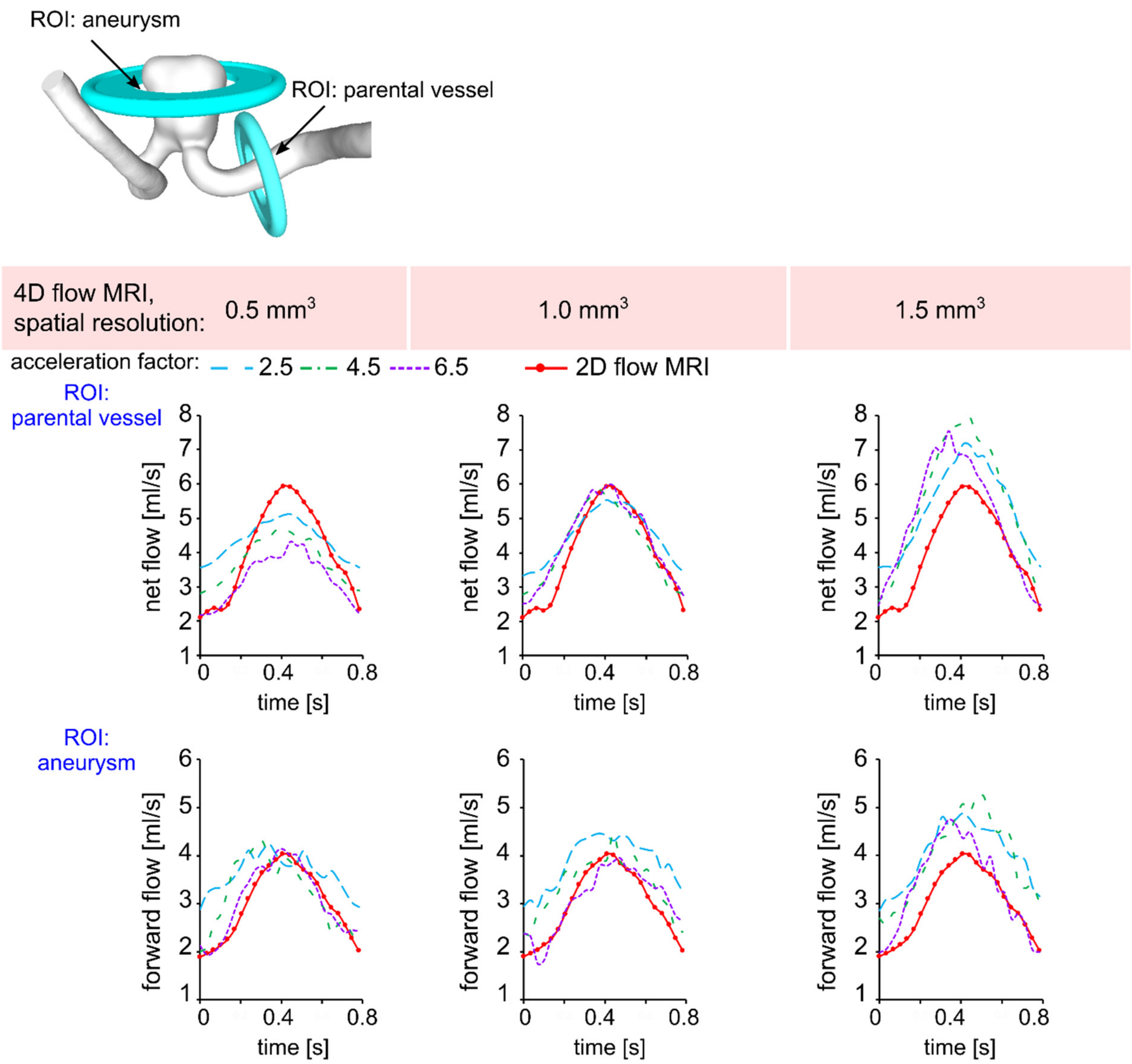

Figure S6. Time-resolved flow rate curves measured with 2D flow and 4D flow MRI in patient-specific aneurysm model. The flow rates were calculated in two ROIs: parental vessel and aneurysm (top). Qualitatively, the peak flow values were overestimated for lower resolution 4D flow data in comparison to the 2D flow.

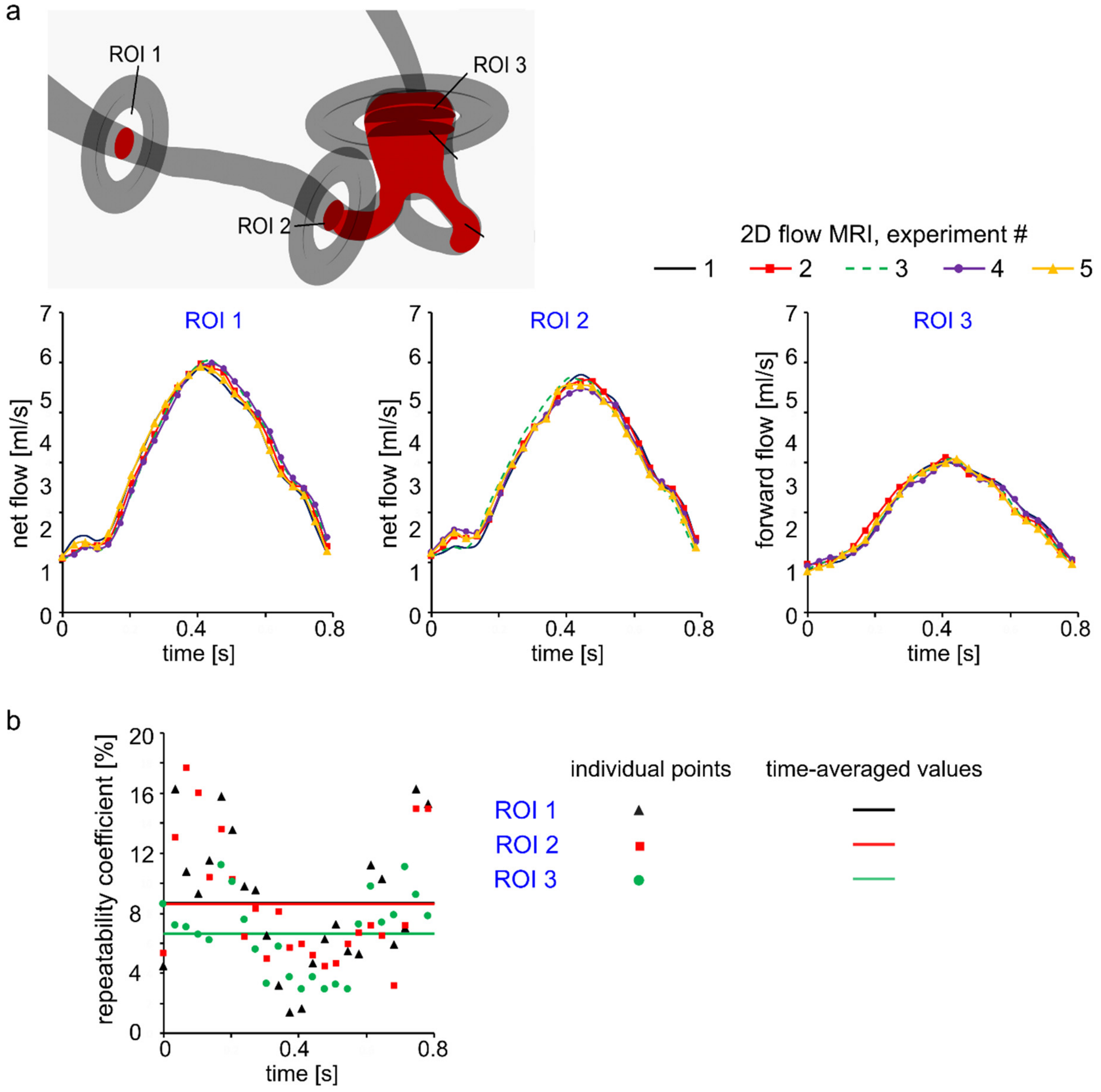

Figure S7. Time-resolved flow rate curves measured with 2D flow MRI at ROI 1-3 (a top) of an aneurysm model. 2D flow MRI experiment was measured five times (a bottom). Note, that only slight variations were observed. Then the repeatability coefficient (RC) was calculated as $RC = 1.96 * \sqrt{2} * SD * 100\%/mean$, where SD and mean values were obtained over flow rate values measured with five consequent 2D flow MRI sequences (b).
